# Online Self-Management Support for Family Caregivers Dealing With Behavior Changes in Relatives With Dementia (Part 2): Randomized Controlled Trial

**DOI:** 10.2196/13001

**Published:** 2020-02-25

**Authors:** Judith G Huis in het Veld, Bernadette M Willemse, Iris FM van Asch, Rob BM Groot Zwaaftink, Paul-Jeroen Verkade, Jos WR Twisk, Renate Verkaik, Marco M Blom, Berno van Meijel, Anneke L Francke

**Affiliations:** 1 Department of Public and Occupational Health, Amsterdam University Medical Center Amsterdam Public Health Research Institute Vrije Universiteit Amsterdam Amsterdam Netherlands; 2 Netherlands Institute of Mental Health and Addiction Trimbos Institute Utrecht Netherlands; 3 Dutch Alzheimer’s Society Amersfoort Netherlands; 4 The Geriant Foundation Region North of Amsterdam Netherlands; 5 Department of Clinical Epidemiology and Biostatistics, Amsterdam University Medical Center Amsterdam Public Health Research Institute Vrije Universiteit Amsterdam Amsterdam Netherlands; 6 Netherlands Institute for Health Services Research Utrecht Netherlands; 7 Department of Psychiatry, Amsterdam University Medical Center Amsterdam Public Health Research Institute Vrije Universiteit Amsterdam Amsterdam Netherlands; 8 Inholland University of Applied Sciences Amsterdam Netherlands; 9 Parnassia Psychiatric Institute The Hague Netherlands; 10 Academy for Masters in Advanced Nursing Science Utrecht Netherlands

**Keywords:** dementia, family caregivers, self-management, support, intervention

## Abstract

**Background:**

Online contacts with a health professional have the potential to support family caregivers of people with dementia.

**Objective:**

The goal of the research was to study the effects of an online self-management support intervention in helping family caregivers deal with behavior changes of a relative with dementia. The intervention—involving among others personal email contacts with a dementia nurse—was compared with online interventions without these email contacts.

**Methods:**

A randomized controlled trial was conducted with 81 family caregivers of people with dementia who live at home. Participants were randomly assigned to a (1) major self-management support intervention consisting of personal email contacts with a specialist dementia nurse, online videos, and e-bulletins; (2) medium intervention consisting only of online videos and e-bulletins; or (3) minor intervention consisting of only the e-bulletins. The primary outcome was family caregivers’ self-efficacy in dealing with behavior changes of the relative with dementia. Secondary outcomes were family caregivers’ reports of behavior problems in the people with dementia and the quality of the relationship between the family caregiver and the person with dementia. Measurements were performed at the baseline and at 6 (T1) and 12 weeks (T2) after the baseline. A mixed-model analysis was conducted to compare the outcomes of the 3 intervention arms.

**Results:**

Family caregivers participating in the major intervention involving email contacts showed no statistically significant differences in self-efficacy after the intervention compared with the minor intervention involving only e-bulletins (difference –0.02, *P*=.99). In the adjusted analysis, the medium intervention (involving videos and e-bulletins) showed a negative trend over time (difference –4.21, *P*=.09) and at T1 (difference –4.71, *P*=.07) compared with the minor intervention involving only e-bulletins. No statistical differences were found between the intervention arms in terms of the reported behavior problems and the quality of the relationship between the family caregiver and the person with dementia.

**Conclusions:**

The expectation that an online self-management support intervention involving email contacts would lead to positive effects and be more effective than online interventions without personal email contacts was not borne out. One explanation might be related to the fact that not all family caregivers who were assigned to that intervention actually made use of the opportunity for personal email contact. The online videos were also not always viewed. To obtain more definite conclusions, future research involving extra efforts to reach higher use rates is required.

**Trial Registration:**

Netherlands Trial Registry NTR6237; http://www.trialregister.nl/trialreg/admin/rctview.asp?TC=6237 (Archived by WebCite at http://www.webcitation.org/6v0S4fxTC)

**International Registered Report Identifier (IRRID):**

RR2-10.2196/resprot.8365

## Introduction

Most people with dementia live at home, and they are often supported by family members who show great dedication in their care [1]. Even so, family care can be a great burden [2], for instance because dealing with behavior changes of relatives is stressful for family caregivers [3]. Changes in behavior can include dependent, aggressive, and suspicious behavior; apathy or indifference; restlessness at night; and masking behavior (hiding the fact that you do not remember things or are unable to do things anymore). These behavior changes are challenging as they often cause distress to family caregivers and/or the person with dementia and adversely affect the quality of life of at least one of the parties [4]. A Dutch nationwide survey found that about 3 in 4 family caregivers of people with dementia experienced problems dealing with changes in their relative’s behavior or mood, in both the initial and the later stages of the disease [5].

Self-management refers to individuals’ ability to manage the symptoms, treatment, physical and psychosocial consequences, and lifestyle inherent in living with a chronic disease. In dementia care, self-management often involves the family caregivers [6]. In addition to caring for their relative, family caregivers must also deal with their own health and the consequences of dementia in their lives [7]. Supporting people in decisions and actions that promote self-management is called self-management support. An increasing number of self-management support interventions have been developed to help family caregivers [8] (eg, in dealing with their relative’s behavior changes). Some of these are Web-based [8]. Using online interventions offers the possibility of getting access to help at any time at any place, without leaving the person with dementia alone [9].

Systematic reviews suggest that online support might have positive effects on the self-efficacy and other psychological or psychosocial outcomes for family caregivers [9-12].

Family caregivers could benefit from multicomponent online interventions combining information and tailored caregiving strategies [10]. In particular, family caregivers might benefit from additional personal online contact with health professionals [10,13] as health professionals can help them apply generic information to their specific situation [14] and give tailored advice based on their needs. Although studies including online professional support have been developed and evaluated, most of them are aimed at general caregiving issues [15-18] and their overall quality of evidence is low [13]. Further research is required to clarify the necessity of personal contacts with a professional [17] for a family caregiver when coping with behavior changes in their relative with dementia.

The aim of this study is to assess whether (1) a major multicomponent intervention, consisting of email contacts with a specialized dementia nurse, videos, and e-bulletins, is more effective than interventions without personal contacts and (2) a medium intervention including videos and e-bulletins is more effective than a minor intervention including e-bulletins only.

The effectiveness of the major and medium interventions was determined by measuring changes in (1) self-efficacy of family caregivers in managing behavior changes of their relative with dementia, (2) behavior problems in the people with dementia, as reported by family caregivers, and (3) quality of the relationship between the family caregiver and the person with dementia.

## Methods

A 3-arm randomized controlled trial (RCT) was carried out between March and August 2017 in the Netherlands. The study is registered in the Netherlands Trial Registry [NTR6237]. The study protocol is published elsewhere [19]. Along with the RCT, a mixed-method process evaluation was performed to evaluate the online self-management support intervention in terms of usability and satisfaction [19].

### Design, Intervention Arms, and Elements

To answer the research questions, a 3-arm RCT was performed with repeated measurements at 3 time points. The 3 intervention arms all focused on helping family caregivers deal with behavior changes in their relative with dementia but varied in the number of elements involved. The intervention arms are referred to as the major, medium, and minor intervention arms. The intervention arms are described elsewhere in more detail [19]. The major intervention arm consisted of the following:

- Family caregivers received 3 personal email contacts with a specialist dementia nurse (in a period of 12 weeks). The nurse supported the family caregivers in managing behavior changes by giving feedback on assignments and tailoring support to the personal needs and questions of the family caregivers. Nurses were trained in a 1-day course in which the intervention was further explained by two of the researchers (JGH and IA). A peer-review session, in which all nurses who provided the intervention participated, took place halfway through the study period. In this peer-review session, the nurses reflected together on the online support they had given.
- Family caregivers received links to 6 online videos with assignments about different types of behavior changes and could choose how many videos they watched and assignments they completed.
- Family caregivers received 6 e-bulletins containing practical information about different types of changes in behavior and how to manage them.

The medium intervention arm consisted only of the online videos and e-bulletins, and the minor intervention arm consisted only of the e-bulletins. For more details, the readers are referred to the full intervention protocol [20].

### Inclusion and Randomization

Family caregivers were eligible to participate in the study if they were at least 18 years old, were a partner or relative of a person diagnosed with dementia who lives at home, had contact with the person with dementia at least once a week, had access to the internet, and gave online consent. Family caregivers were recruited via the Dutch Alzheimer Society’s panel, the Dutch Alzheimer Society’s online forum (with 7000 monthly visitors), the Dementie.nl website [21], and the Dutch Alzheimer Society’s social media accounts (Twitter and Facebook). Details of the recruitment procedure have been described elsewhere [19].

After online consent was given (see the study protocol for more detail [19]), family caregivers were randomly allocated by a researcher (JGH) to 1 of the 3 intervention arms using a randomization schedule. Block randomization was applied to achieve an equal likelihood of the participant being allocated to each of the 3 intervention arms [22]. An independent epidemiologist prepared the randomization schedule using several block sizes of 6 and 9.

Participants could not be blinded as it is impossible to blind participants to the sort of eHealth intervention they are receiving [23].

### Sample Size

In this study, we expected that (1) both the major and medium intervention arms would lead to a greater improvement in self-efficacy than the minor intervention arm and that (2) the major intervention arm would show larger improvements in self-efficacy than the medium intervention arm.

In another study, large effect sizes were found for self-efficacy in family caregivers with dementia [24]. Based on a difference of 0.8 standard deviation units between the groups and assuming a significance level of 5%, a power of 80%, and correlation of .60 between the two repeated measures, the number of subjects needed per group was 20. Taking into account a dropout rate of 20%, 24 participants per group were needed.

Another consideration was that the specialist dementia nurses had limited previous experience in providing self-management support through email contacts. We therefore expected a learning curve for the dementia nurses during the study, which might also have had consequences for the measured effects on family caregivers. Following the randomization schedule, one additional block of 9 participants (3 in each group) was added to the sample so that we could take a brief learning curve into account. This brought the total number of participants that had to be recruited to 81.

### Measurement Procedures

Measurements were performed at 3 points in time: (T0) baseline assessment, (T1) 6 weeks after the baseline assessment, and (T2) 12 weeks after the baseline assessment. Measurements were done by online questionnaires administered to the participating family caregivers through an email link. After 1 and 2 weeks, participants were reminded (if needed) to complete the questionnaires.

### Primary Outcome

The primary outcome variable (self-efficacy) was measured using the Trust in Our Own Abilities (TRUST) instrument, a questionnaire in Dutch. The questionnaire had been used previously to measure self-efficacy in family caregivers of people with dementia living at home [25]. The TRUST questionnaire has 32 items divided into 3 subscales: solution orientation (8 items), resilience (15 items), and proactive competence (9 items). For this study, one item from the original 37-item TRUST questionnaire was added as this item reflected the main goal of this intervention. This item was queried as “How well can you, in your own opinion, deal with changed behavior of your relative, such as aggression, apathy, and dependence?” (translated from Dutch). Since the TRUST questionnaire is quite new and has only been validated and tested with pilot data, a principal component analysis was performed. A total of 33 items were tested in a principal component analysis. All 33 items were loading on the same factor. However, 4 of the 33 items were not loading strongly enough (cutoff point <0.4) [26]. When these items were dropped, the Cronbach alpha for our sample was .925. Only the revised sum score (29 items) will therefore be studied. Items ranged from 0=not at all to 4=very good). The higher the score, the greater the perceived competence in caring for someone with dementia [25].

### Secondary Outcomes

The first secondary outcome variable was the presence and reaction scores for mood and behavior problems, measured using the Dutch version of the Revised Memory and Behavioral Problem Checklist (RMBPC) [27,28]. The RMBPC is a self-assessment questionnaire that can be broken down into scales for disruptive behavior (8 items), depression (9 items), and memory-related problems (7 items). Overall reliability for this scale is .84 for patient behavior and .90 for caregiver reaction [27].

For this study, only disruptive behavior will be studied as this was the outcome of interest. Family caregivers were asked to rate the occurrence of specific behavior on a scale from 0 to 4 (0=never, 1=rarely, 2=regularly, 3=often, 4=always) and parallel their reaction scores for the degree of distress (0=not upset, 1=not very upset, 2=quite upset, 3=extremely upset).

The mean scores of the occurrence of behavior and family caregivers’ reaction to these problems were calculated. For behaviors that did not occur, a reaction score of 0 (not upset) was assigned [29].

A second secondary outcome variable concerned the positive and negative aspects of the relationship between the person with dementia and the family caregiver. This was measured by the Dyadic Relationship Scale (DRS). The family caregiver version has 11 items in 2 subscales: dyadic strain (5 items) and positive dyadic interaction (6 items). Family caregivers were asked to rate the separate items on a 4-point scale (1=strongly disagree, 2=disagree, 3=agree, 4=strongly agree). Overall reliability for this scale is .89 for negative dyadic strain and .85 for positive dyadic interaction [30].

### Analyses

All data were analyzed using SPSS Statistics version 22.0 (IBM Corp). Mixed-model analyses were carried out to compare primary and secondary outcomes between the major and minor intervention arm and between the medium and minor intervention arm over time and at T1 and T2. Mixed-model analyses were performed to take into account the correlation between the 2 repeated measurements within the subject (T1 and T2). To obtain the intervention effect at 2 different time points, time and interaction between intervention and time were added to the model. All mixed-model analyses were adjusted for the baseline value of the particular outcome. In addition to crude effects, effects adjusted for gender, type of relationship, appearance of first symptoms, education level, and shared caregiving were also estimated.

### Ethics Procedures

The study was approved by the VU University Medical Center’s Medical Ethics Committee (reference 2016.559). It had no objections to the study. All participants were required to give their informed consent for participation via an online informed consent form. Only the research team members had access to the data. Agreements about how to archive, share, and store data were signed by the organizations responsible for collecting the data.

## Results

### Participant Characteristics

A total of 158 family caregivers expressed interest in participating in the study. After sending an information letter, the first 81 caregivers who signed the online informed consent form and completed the baseline assessment were included.

**Figure 1:**
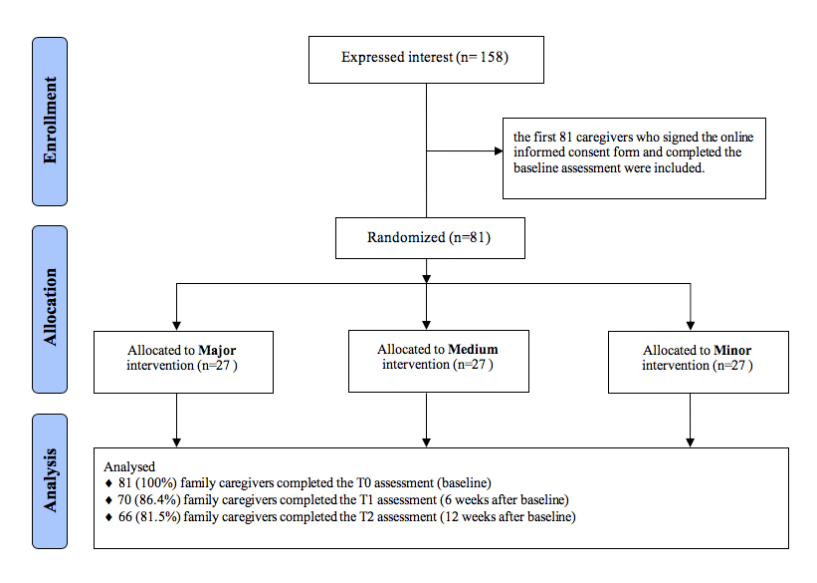
Study flowchart based on the Consolidated Standard of Reporting Trials flow diagram [29].

After completing the baseline questionnaire, participants were randomly allocated to the major (27), medium (27), or minor (27) intervention arms following the block randomization schedule (Figure 1) [31]. A total of 86% (70/81) of family caregivers completed the T1 assessment (6 weeks after baseline), and 82% (66/81) of family caregivers completed the T2 assessment (12 weeks after baseline).

Baseline data for the caregivers included are listed in Table 1. At baseline, family caregivers were on average aged 56.5 (SD 12.5) years (range 23-80 years), primarily female (71/81, 88%), and half of them had completed a professional or academic degree (40/81, 49%). The relatives with dementia they were caring for were mostly their mother or father (or a parent-in-law) (46/81, 57%) or their partner (32/81, 40%). The individuals with dementia were on average aged 75.1 (SD 9.9) years (range 49-96 years) and more often male (42/81, 52%), with Alzheimer disease being the most prevalent form of dementia (47/81, 57%). In most cases, the first symptoms of dementia had appeared 4 years or more previously (42/81, 52%). Behaviors that family caregivers had the most difficulty dealing with were dependent (22/81, 27%) and masking behavior (19/81, 24%). At baseline, most family caregivers stated that they were somewhat (35/81, 43%) or significantly (31/81, 38%) burdened by the care for their relative with dementia.

**Table 1:** Baseline data for the caregivers included (N=81).

Characteristics		Value
**Group, n (%)**		
	Major	27 (33)
	Medium	27 (33)
	Minor	27 (33)
Gender of family caregiver, female, n (%)		71 (88)
Age of family caregiver, mean (SD) range		56.5 (12.5) 23-80
Gender of person with dementia, female, n (%)		39 (48)
Age of person with dementia, mean (range; SD)		75.1 (9.9) 49-96
**Relationship of family caregiver to person with dementia, n (%)**		
	Partner	32 (40)
	Adult child (son/daughter or son-in-law/daughter-in-law)	46 (57)
	Other family member	3 (4)
Person with dementia has their own household, n (%)		25 (31)
Same household as person with dementia, n (%)		33 (41)
**First symptoms of dementia (according to the family caregiver), n (%)**		
	<2 years	15 (19)
	2 to 4 years	24 (30)
	>4 years or more	42 (52)
**Type of dementia of the relative with dementia, n (%)**		
	Alzheimer disease	47 (57)
	Vascular dementia	13 (16)
	Frontotemporal dementia	3 (4)
	Dementia with Lewy bodies	2 (3)
	Mixed dementia	9 (11)
	Not known	7 (9)
**Highest educational attainment, n (%)**		
	Primary school	8 (10)
	High school (preparatory to vocational education) and vocational training	17 (21)
	Professional or academic/university	40 (49)
	Missing	16 (20)
**Burden (at baseline), n (%)**		
	Barely	6 (7)
	Somewhat	35 (43)
	Fairly	31 (38)
	High	9 (11)
**Behavior that family caregiver has the most difficulty dealing with, n (%)**		
	Dependent behavior	22 (27)
	Aggressive behavior	9 (11)
	Suspicious behavior	12 (15)
	Apathy or indifference	9 (11)
	Nighttime restlessness	10 (12)
	Masking behavior	19 (24)

### Sensitivity Analyses

The initial analyses were performed without the first randomized 9 caregivers (who were the learning curve block). These initial analyses among 72 family caregivers revealed no differences with analyses of data for the overall group of 81 family caregivers. The final analyses were therefore conducted on all 81 randomized family caregivers. Multimedia Appendix 1 and 2 show the results of the mixed-model analyses.

### Effects on Self-Efficacy

**Figure 2:**
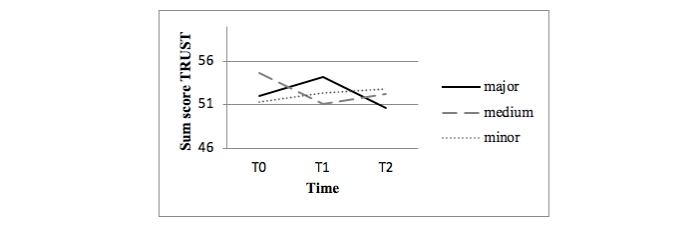
Observed mean scores for the sum score of the Trust in Our Own Abilities questionnaire (29 items, range 0-87).

Figure 2 shows the observed mean scores for the sum score of the TRUST questionnaire. In the mixed-model analyses, the major intervention (involving personal email contacts as well as videos and e-bulletins) did not show significant differences in self-efficacy in both the crude and adjusted analyses compared with the minor intervention arm. Also, no statistical differences were found between the medium intervention (involving videos and e-bulletins) and minor intervention (only involving e-bulletins) in the crude analyses.

However, the medium intervention unexpectedly showed a negative trend over time in the adjusted analyses (difference –4.21, *P*=.09) and at T1 (difference –4.71, *P*=.07) compared with the minor intervention involving e-bulletins only.

### Effects on Behavior Changes in the Relative With Dementia

**Figure 3:**
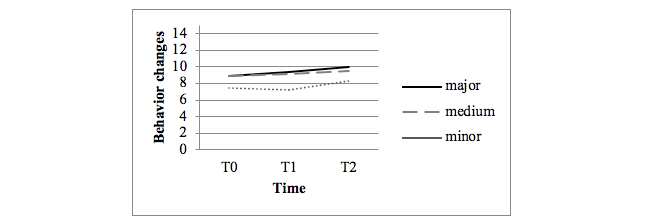
Observed mean scores for behavior changes (disruption subscale of the Revised Memory and Behavioral Problem Checklist questionnaire; 8 items, range 0-32).

**Figure 4:**
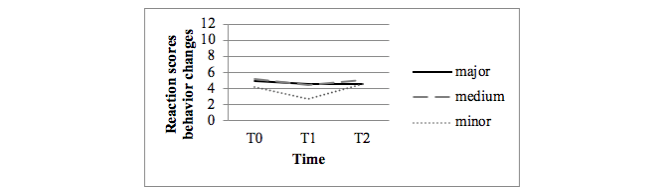
Observed mean scores of family caregivers’ reaction scores for disruptive behavior of their relatives with dementia (disruption subscale of the Revised Memory and Behavioral Problem Checklist questionnaire; 8 items, range 0-24).

Figure 3 shows the observed mean scores for behavior changes in the person with dementia as reported by the family caregivers. Figure 4 shows the observed mean scores for family caregivers’ reaction scores for disruptive behavior (disruption subscale of the RMBPC questionnaire). No statistical differences were found in the crude and adjusted analyses between the major and minor intervention arms or between the medium and minor intervention arms regarding the occurrence of behavior changes.

However, statistical differences were found between the major and minor intervention arms in the adjusted analyses at T1 for the family caregivers’ reaction scores for disruptive behavior (difference 2.02, *P*=.05).

### Effects on the Quality of the Relationship

**Figure 5:**
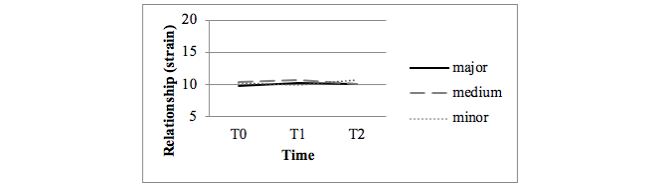
Observed mean scores for the strain in relationships (Dyadic Relationship Scale questionnaire; 5 items, range 5-20).

**Figure 6:**
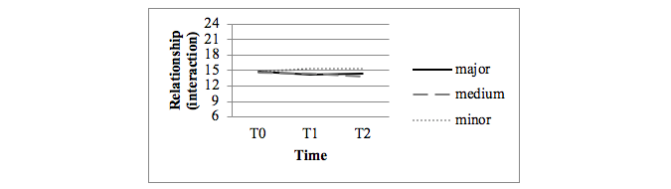
Observed mean scores for interaction in relationships (Dyadic Relationship Scale questionnaire; 6 items, range 6-24).

Figures 5 and 6 display the observed mean scores for the DRS questionnaire subscales Strain and Interaction. No statistical differences were found in the quality of the relationship in both the crude and adjusted analyses between the major and minor intervention arms and the medium and minor intervention arms at all measurements (over time, at T1 and T2).

## Discussion

### Principal Findings

Online self-management support involving email contacts with a specialist dementia nurse, videos, and e-bulletins showed no significant difference in family caregivers’ self-efficacy compared with online interventions not involving personal email contacts. Furthermore, no measurable improvements could be found for the medium intervention involving online videos and e-bulletins compared with the minor intervention only involving e-bulletins.

In addition, no differences were found between the online intervention arms for the quality of the relationship between the person with dementia and the family caregiver and the occurrence of behavior changes. These results are contrary to our expectation that family caregivers who received email support would be better assisted in dealing with and responding to changes in behavior and would therefore improve in terms of self-efficacy. We expected that increased self-efficacy and better response of the family caregiver would also have an effect on the person with dementia and would therefore result in less strain on the relationship, better interaction, and n decrease in the occurrence of behavior changes. However, as no effect on self-efficacy was found, this could also explain why no effect could be detected on the secondary outcomes (quality of the relationship and the occurrence of behavior changes) in this study.

Moreover, the medium arm (consisting of video and e-bulletins) showed a negative trend in family caregivers’ self-efficacy over time and shortly after the intervention (at T1). One possible explanation may be that the online videos made family caregivers more aware of how they were dealing with behavioral changes of their relative with dementia. This understanding—obtained from watching the online videos—may have influenced their confidence in their ability to successfully influence behavioral changes. This only seems to affect family caregivers at the moment of watching the video (6 weeks after baseline) and did not remain after a longer period of time (12 weeks after baseline).

This negative trend regarding family caregivers’ self-efficacy was not observed in the major arm, even though those participants shared the same experience of the online videos with the medium arm. Perhaps the personal email contacts with the nurse in the major arm were enough to offset a negative effect of increased awareness through the videos but not enough to have a positive effect on the measured self-efficacy.

An explanation for the lack of improvement in self-efficacy could be that family caregivers were not able to translate the information and advice to their personal situations [7] despite the fact that in the major intervention arm, the dementia nurses tried to tailor their email contacts to the individual situation of the family caregiver. Also, the mean scores at baseline for self-efficacy, behavior changes, and relationships were already quite good. As a result, there might have been less room for improvements.

Contrary to our expectations, it was found that family caregivers in the major intervention arm were significantly more distressed at T1 by the disruptive behavior of their relatives with dementia than family caregivers who only received e-bulletins. An explanation for this can be that, initially, a more intensive and major intervention (involving personal email contacts, videos, and ebulletins) sharpened caregivers’ focus on behavioral changes in their relative with dementia. This initially might have increased awareness, which may have led to an increased report of distress shortly after the intervention at T1. However, there was no statistical difference between these two groups at T2, a more distance time point.

Along with the RCT presented in this paper, a process evaluation was carried out [32]. The process evaluation showed that the personal contacts with the nurse were highly valued and believed to add value to the online videos and e-bulletins. Nonetheless, these qualitative results were not reflected in the quantitative results in this paper.

The process evaluation also gave some additional explanations for the unexpected results in the RCT. First, the process evaluation showed variation in the extent to which family caregivers made use of the various elements. Of the family caregivers in question, 78% used the opportunity of having email contacts and 80% clicked on the links to one or more videos but just 37% of all family caregivers clicked on the links of at least one e-bulletin. Also, the use of email contacts, videos, and/or e-bulletins varied considerably within in each group. Therefore, the distinction between the 3 intervention arms became less, which makes it less likely to find statistically significant differences between the intervention arms. Low use rates and differences in the use of online interventions are known problems [33,34] that could explain why no positive effects were found in this study.

Second, both family caregivers and nurses mentioned that the email contacts helped family caregivers share their stories about their experiences with the changing behavior of their relative with dementia. The email contacts seemed therefore less focused on finding ways to deal with behavioral changes. Although receiving appreciation and acknowledgment is essential for family caregivers [35], this could explain why our study found no effects on self-efficacy, measured behavior, or quality of the relationship.

Last, positive effects could be left out because the participants already knew a lot about dementia and how to deal with behavioral changes of their relative. According to the dementia nurses, the participants involved were mainly family caregivers who were already consciously engaged in collecting information about dementia. These family caregivers all had internet access and were often relatively young and well educated. This group had previously gained information and advice about coping with behavioral changes, which might explain the lack of positive effects on self-efficacy.

Based on the findings of the process evaluation [32], we have 2 recommendations for future use of the intervention. First, we recommend that nurses are instructed more explicitly and made more aware of the importance of the integrated use of the various elements (email contacts, videos, and e-bulletin) in the interventions. Second, for future use the intervention could involve more email contacts.

### Strengths and Limitations

Several strengths of this study can be noted. First, the online component of this study helped provide accessible and tailored support for family caregivers. Caregivers could participate nationwide and use the online assistance at times that suited them. Second, selective dropout was reduced by using a mixed-model analysis that also included incomplete cases (ie, participants who did not complete the online questionnaire either at the 6- or 12-week follow-up). Finally, selection bias was reduced by using a prepared randomization schedule to randomly allocate family caregivers to 1 of the 3 intervention arms [22].

However, some limitations of this study are worth mentioning. First, in the power calculation, we had estimated a difference of 0.8 between the intervention arms to detect a significant effect of the major self-management support intervention compared with the other intervention arms. The estimated difference proved to have been an overestimate. The small sample size might therefore have played a part in the null findings for our hypothesis that the major intervention arm would have a greater effect on self-efficacy than the other intervention arms. We acknowledge that our study may have been underpowered for detecting an effect of the online self-management support intervention. For future studies, larger studies may be required to establish the effectiveness of online self-management support interventions [36].

Second, due to the small sample size, we were unable to determine the effects on participants who actually used the intervention components. Instead, data of all included participants were analyzed. Future research should focus on which intervention components best fit specific family caregivers. It is important to determine the family caregivers who will benefit the most from additional online assistance in order to provide tailored, personalized support. This will be more cost effective, allowing nurses’ support to be offered to the people who need it the most.

### Conclusion

The online self-management support intervention involving email contacts did not lead to positive effects compared with online interventions without personal email contacts. Furthermore, the medium intervention involving online videos and e-bulletins showed no statistical improvements compared with the minor intervention involving e-bulletins only. To come to more definitive conclusions, future research involving extra efforts to achieve high use rates is required.

## Appendix

Multimedia Appendix 1 Results for the major intervention arm compared with the minor intervention arm over time, at T1 and T2, on the outcomes Trust in Our Own Abilities, Revised Memory and Behavioral Problem Checklist, and Dyadic Relationship Scale questionnaires.

Multimedia Appendix 2 Results for the medium intervention arm compared with the minor intervention arm over time, at T1 and T2, on the outcomes Trust in Our Own Abilities, Revised Memory and Behavioral Problem Checklist, and Dyadic Relationship Scale questionnaires.

Multimedia Appendix 3 CONSORT‐EHEALTH checklist (V.1.6.1).

## Abbreviations

DRS: Dyadic Relationship Scale
RCT: randomized controlled trial
RMBPC: Revised Memory and Behavioral Problem Checklist
TRUST: Trust in Our Own Abilities

## References

[1] Wimo A, Prince M (2010). Alzheimer's Disease International.

[2] Cheng S (2017). Dementia caregiver burden: a research update and critical analysis. Curr Psychiatry Rep.

[3] Chiao C, Wu H, Hsiao C (2015). Caregiver burden for informal caregivers of patients with dementia: a systematic review. Int Nurs Rev.

[4] Feast A, Orrell M, Charlesworth G, Melunsky N, Poland F, Moniz-Cook E (2016). Behavioural and psychological symptoms in dementia and the challenges for family carers: systematic review. Br J Psychiatry.

[5] Zwaanswijk M, Peeters JM, van Beek AP, Meerveld JH, Francke AL (2013). Informal caregivers of people with dementia: problems, needs and support in the initial stage and in subsequent stages of dementia: a questionnaire survey. Open Nurs J.

[6] Barlow J, Wright C, Sheasby J, Turner A, Hainsworth J (2002). Self-management approaches for people with chronic conditions: a review. Patient Educ Couns.

[7] Huis in het Veld J, Verkaik R, van Meijel B, Verkade P, Werkman W, Hertogh C, Francke A (2016). Self-management by family caregivers to manage changes in the behavior and mood of their relative with dementia: an online focus group study. BMC Geriatr.

[8] Huis in het Veld JG, Verkaik R, Mistiaen P, van Meijel B, Francke AL (2015). The effectiveness of interventions in supporting self-management of informal caregivers of people with dementia: a systematic meta review. BMC Geriatr.

[9] Parra-Vidales E, Soto-Pérez F, Perea-Bartolomé MV, Franco-Martín MA, Muñoz-Sánchez JL (2017). Online interventions for caregivers of people with dementia: a systematic review. Actas Esp Psiquiatr.

[10] Boots LMM, de Vugt ME, van Knippenberg RJM, Kempen GIJM, Verhey FRJ (2014). A systematic review of Internet-based supportive interventions for caregivers of patients with dementia. Int J Geriatr Psychiatry.

[11] Waller A, Dilworth S, Mansfield E, Sanson-Fisher R (2017). Computer and telephone delivered interventions to support caregivers of people with dementia: a systematic review of research output and quality. BMC Geriatr.

[12] Jackson D, Roberts G, Wu ML, Ford R, Doyle C (2016). A systematic review of the effect of telephone, internet or combined support for carers of people living with Alzheimer's, vascular or mixed dementia in the community. Arch Gerontol Geriatr.

[13] Hopwood J, Walker N, McDonagh L, Rait G, Walters K, Iliffe S, Ross J, Davies N (2018). Internet-based interventions aimed at supporting family caregivers of people with dementia: systematic review. J Med Internet Res.

[14] Blom MM, Bosmans JE, Cuijpers P, Zarit SH, Pot AM (2013). Effectiveness and cost-effectiveness of an internet intervention for family caregivers of people with dementia: design of a randomized controlled trial. BMC Psychiatry.

[15] Bass DM, McClendon MJ, Brennan PF, McCarthy C (1998). The buffering effect of a computer support network on caregiver strain. J Aging Health.

[16] Kwok T, Au A, Wong B, Ip I, Mak V, Ho F (2014). Effectiveness of online cognitive behavioral therapy on family caregivers of people with dementia. Clin Interv Aging.

[17] Blom MM, Zarit SH, Groot ZR, Cuijpers P, Pot AM (2015). Effectiveness of an Internet intervention for family caregivers of people with dementia: results of a randomized controlled trial. PLoS One.

[18] Chiu T, Marziali E, Colantonio A, Carswell A, Gruneir M, Tang M, Eysenbach G (2009). Internet-based caregiver support for Chinese Canadians taking care of a family member with alzheimer disease and related dementia. Can J Aging.

[19] Huis In Het Veld JG, Willemse BM, van Asch IF, Groot Zwaaftink RB, Verkade P, Veldhuijzen NJ, Verkaik R, Blom MM, Francke AL (2017). Online self-management support for family caregivers to help them manage behavior changes in their relative with dementia: study protocol for a randomized controlled trial and a process evaluation. JMIR Res Protoc.

[20] Online self-management support for informal caregivers for managing behavioral changes in their relative with dementia: intervention protocol.

[21] Dementie.

[22] Efird J (2011). Blocked randomization with randomly selected block sizes. Int J Environ Res Public Health.

[23] Baker TB, Gustafson DH, Shaw B, Hawkins R, Pingree S, Roberts L, Strecher V (2010). Relevance of CONSORT reporting criteria for research on eHealth interventions. Patient Educ Couns.

[24] Boots LM, de Vugt ME, Kempen GI, Verhey FR (2018). Effectiveness of a blended care self-management program for caregivers of people with early-stage dementia (partner in balance): randomized controlled trial. J Med Internet Res.

[25] Wijma EM, Veerbeek MA, Prins M, Pot AM, Willemse BM (2018). A virtual reality intervention to improve the understanding and empathy for people with dementia in informal caregivers: results of a pilot study. Aging Ment Health.

[26] Field A (2013). Discovering Statistics Using IBM SPSS Statistics.

[27] Teri L, Truax P, Logsdon R, Uomoto J, Zarit S, Vitaliano PP (1992). Assessment of behavioral problems in dementia: the revised memory and behavior problems checklist. Psychol Aging.

[28] Teunisse S, De Haan R, Walstra G, De Rooij S, Zwart M (1997). Behavioural problems in mild dementia: clinical relevance and methodological evaluation of the revised memory and behavioural problems checklist [Thesis].

[29] Prick A, de Lange J, Twisk J, Pot AM (2015). The effects of a multi-component dyadic intervention on the psychological distress of family caregivers providing care to people with dementia: a randomized controlled trial. Int Psychogeriatr.

[30] Sebern MD, Whitlatch CJ (2007). Dyadic relationship scale: a measure of the impact of the provision and receipt of family care. Gerontologist.

[31] Moher D, Schulz KF, Altman DG (2001). The CONSORT statement: revised recommendations for improving the quality of reports of parallel-group randomised trials. Lancet.

[32] Huis In Het Veld JG, van Asch IFM, Willemse BM, Verkade P, Pot AM, Blom MM, Groot Zwaaftink RBM, Francke AL (2019). Process evaluation of nurse-led online self-management support for family caregivers to deal with behavior changes of a relative with dementia (part 1): mixed methods study. J Med Internet Res.

[33] Eysenbach G (2013). CONSORT-EHEALTH: implementation of a checklist for authors and editors to improve reporting of web-based and mobile randomized controlled trials. Stud Health Technol Inform.

[34] Wangberg SC, Bergmo TS, Johnsen JK (2008). Adherence in internet-based interventions. Patient Prefer Adherence.

[35] Huis In Het Veld JG, Verkaik R, van Meijel B, Verkade P, Werkman W, Hertogh CM, Francke AL (2018). Self-management support and ehealth when managing changes in behavior and mood of a relative with dementia: an asynchronous online focus group study of family caregivers' needs. Res Gerontol Nurs.

[36] Cristancho-Lacroix V, Wrobel J, Cantegreil-Kallen I, Dub T, Rouquette A, Rigaud A (2015). A web-based psychoeducational program for informal caregivers of patients with Alzheimer's disease: a pilot randomized controlled trial. J Med Internet Res.

