# Supplementary material for: Online Self-Management Support for Family Caregivers Dealing With Behavior Changes in Relatives With Dementia (Part 2): Randomized Controlled Trial
Source: J Med Internet Res. 2020 Feb 25;22(2):e13001. doi: 10.2196/13001 (PMC7064946; doi:10.2196/13001)
Supplement: Multimedia Appendix 1 [file jmir_v22i2e13001_app1.docx]

**Multimedia Appendix 1. Results for the major intervention arm compared to the minor intervention arm over time, at T1 and at T2 on the outcomes TRUST, RMBPC and DRS.**

| **MAJOR INTERVENTION ARM** | | | | | | | | | | | | | | | | | | | | | | | | |
| --- | --- | --- | --- | --- | --- | --- | --- | --- | --- | --- | --- | --- | --- | --- | --- | --- | --- | --- | --- | --- | --- | --- | --- | --- |
|  | **Analyses over Time** | | | | | | | | **Analyses at T1** | | | | | | | | **Analyses at T2** | | | | | | | |
|  | Crude analysis^a^ | | | | Adjusted analysis^b^ | | | | Crude analysis^a^ | | | | Adjusted analysis^b^ | | | | Crude analysis^a^ | | | | Adjusted analysis^b^ | | | |
|  | B | P value | 95%CI | | B | P value | 95%CI | | B | P value | 95%CI | | B | P value | 95%CI | | B | P value | 95%CI | | B | P value | 95%CI | |
| TRUST | -0.02 | .99 | -4.16 | 4.12 | -1.12 | .65 | -5.98 | 3.74 | 1.97 | .40 | -2.65 | 6.59 | 0.30 | .91 | -5.14 | 5.73 | -2.16 | .36 | -6.86 | 2.53 | -2.40 | .38 | -7.77 | 2.97 |
| RMBPC *Dis* | 0.35 | .71 | -1.56 | 2.27 | 0.60 | .59 | -1.62 | 2.83 | 0.58 | .59 | -1.55 | 2.70 | 1.06 | .40 | -1.42 | 3.54 | 0.12 | .91 | -2.04 | 2.27 | 0.13 | .93 | -2.33 | 2.59 |
| RMBPC-R *Dis* | 0.59 | .42 | -0.88 | 2.06 | 1.09 | .21 | -0.63 | 2.81 | 1.22 | .16 | -0.49 | 2.94 | 2.02 | .05 | 0.04 | 4.00 | -0.08 | .93 | -1.81 | 1.65 | 0.14 | .89 | -1.84 | 2.12 |
| DRS-S | 0.33 | .55 | -0.76 | 1.41 | 0.24 | .70 | -1.02 | 1.52 | 0.56 | .37 | -0.68 | 1.79 | 0.41 | .57 | -1.05 | 1.87 | 0.08 | .90 | -1.18 | 1.34 | 0.06 | .93 | -1.38 | 1.51 |
| DRS-I | -0.46 | .47 | -1.72 | 0.80 | -0.37 | .62 | -1.86 | 1.11 | -0.39 | .59 | -1.84 | 1.06 | -0.35 | .69 | -2.05 | 1.36 | -0.53 | .47 | -2.00 | 0.93 | -0.37 | .66 | -2.05 | 1.31 |

^a^Adjusted for the baseline value of the outcome variable.

^b^Adjusted for the baseline value of the outcome variable, gender, type of relationship, appearance of first symptoms, education level and shared caregiving

Abbreviations: TRUST, revised total score for Trust in Own Abilities (29 items); RMBPC, Revised Memory and Behavioral Problem Checklist; Dis, Disruptive behavior; RMBPC-r, RMBPC-R Dis, family caregivers’ reaction scores for disruptive behavior of the relative with dementia; DRS-S, Dyadic Relationship Scale - Strain; DRS-I, Dyadic Relationship Scale - Interaction.
