# Supplementary material for: Online Self-Management Support for Family Caregivers Dealing With Behavior Changes in Relatives With Dementia (Part 2): Randomized Controlled Trial
Source: J Med Internet Res. 2020 Feb 25;22(2):e13001. doi: 10.2196/13001 (PMC7064946; doi:10.2196/13001)
Supplement: Multimedia Appendix 2 [file jmir_v22i2e13001_app2.docx]

**Multimedia Appendix 2. Results for the medium intervention arm compared to the minor intervention arm over time, at T1 and at T2 on the outcomes TRUST, RMBPC and DRS**

| **MEDIUM INTERVENTION ARM** | | | | | | | | | | | | | | | | | | | | | | | | |
| --- | --- | --- | --- | --- | --- | --- | --- | --- | --- | --- | --- | --- | --- | --- | --- | --- | --- | --- | --- | --- | --- | --- | --- | --- |
|  | **Analyses over Time** | | | | | | | | **Analyses at T1** | | | | | | | | **Analyses at T2** | | | | | | | |
|  | Crude analysis^a^ | | | | Adjusted analysis^b^ | | | | Crude analysis^a^ | | | | Adjusted analysis^b^ | | | | Crude analysis^a^ | | | | Adjusted analysis^b^ | | | |
|  | B | P value | 95%CI | | B | P value | 95%CI | | B | P value | 95%CI | | B | P value | 95%CI | | B | P value | 95%CI | | B | P value | 95%CI | |
| TRUST | -3.03 | .14 | -7.06 | 1.00 | -4.21 | .09 | -9.01 | 0.60 | -3.04 | .18 | -7.49 | 1.41 | -4.71 | .07 | -9.94 | 0.52 | -2.95 | .20 | -7.52 | 1.63 | -3.63 | .18 | -8.93 | 1.68 |
| RMBPC *Dis* | 0.32 | .73 | -1.54 | 2.18 | 0.96 | .38 | -1.23 | 3.15 | 0.29 | .78 | -1.78 | 2.35 | 1.13 | .35 | -1.25 | 3.52 | 0.37 | .72 | -1.72 | 2.47 | 0.89 | .46 | -1.52 | 3.31 |
| RMBPC-R *Dis* | 0.53 | .46 | -0.88 | 1.95 | 0.49 | .56 | -1.20 | 2.18 | 0.87 | .29 | -0.75 | 2.49 | 1.13 | .23 | -0.76 | 3.02 | 0.21 | .81 | -1.47 | 1.88 | -0.09 | .93 | -2.01 | 1.84 |
| DRS-S | 0.03 | .95 | -1.02 | 1.09 | 0.52 | .40 | -0.70 | 1.75 | 0.54 | .37 | -0.65 | 1.74 | 1.05 | .13 | -0.32 | 2.42 | -0.53 | .39 | -1.76 | 0.69 | -0.53 | .94 | -1.45 | 1.34 |
| DRS-I | -0.76 | .22 | -1.99 | 0.47 | -0.81 | .27 | -2.28 | 0.66 | -0.62 | .38 | -2.01 | 0.77 | -0.84 | .31 | -2.47 | 0.79 | -0.92 | .21 | -2.35 | 0.51 | -0.85 | .31 | -2.51 | 0.81 |

^a^Adjusted for the baseline value of the outcome variable.

^b^Adjusted for the baseline value of the outcome variable, gender, type of relationship, appearance of first symptoms, education level and shared caregiving

Abbreviations: TRUST, revised total score for Trust in Own Abilities (29 items); RMBPC, Revised Memory and Behavioral Problem Checklist; Dis, Disruptive behavior; RMBPC-r, RMBPC-R Dis, family caregivers’ reaction scores for disruptive behavior of the relative with dementia; DRS-S, Dyadic Relationship Scale - Strain; DRS-I, Dyadic Relationship Scale - Interaction.
