# Supplementary material for: Online Self-Management Support for Family Caregivers Dealing With Behavior Changes in Relatives With Dementia (Part 2): Randomized Controlled Trial
Source: J Med Internet Res. 2020 Feb 25;22(2):e13001. doi: 10.2196/13001 (PMC7064946; doi:10.2196/13001)
Supplement: Multimedia Appendix 3 [file jmir_v22i2e13001_app3.pdf]

CONSORT-EHEALTH (V 1.6.1) - Submission/Publication Form

The CONSORT-EHEALTH checklist is intended for authors of randomized trials evaluating web-based and Internet-based applications/interventions, including mobile interventions, electronic games (incl multiplayer games), social media, certain telehealth applications, and other interactive and/or networked electronic applications. Some of the items (e.g. all subitems under item 5 - description of the intervention) may also be applicable for other study designs.

The goal of the CONSORT EHEALTH checklist and guideline is to be
a) a guide for reporting for authors of RCTs,
b) to form a basis for appraisal of an ehealth trial (in terms of validity)

CONSORT-EHEALTH items/subitems are MANDATORY reporting items for studies published in the Journal of Medical Internet Research and other journals / scientific societies endorsing the checklist.

Items numbered 1., 2., 3., 4a., 4b etc are original CONSORT or CONSORT-NPT (non-pharmacologic treatment) items.
Items with Roman numerals (i., ii, iii, iv etc.) are CONSORT-EHEALTH extensions/clarifications.

As the CONSORT-EHEALTH checklist is still considered in a formative stage, we would ask that you also RATE ON A SCALE OF 1-5 how important/useful you feel each item is FOR THE PURPOSE OF THE CHECKLIST and reporting guideline (optional).

Mandatory reporting items are marked with a red *.
In the textboxes, either copy & paste the relevant sections from your manuscript into this form - please include any quotes from your manuscript in QUOTATION MARKS,
or answer directly by providing additional information not in the manuscript, or elaborating on why the item was not relevant for this study.

YOUR ANSWERS WILL BE PUBLISHED AS A SUPPLEMENTARY FILE TO YOUR PUBLICATION IN JMIR AND ARE CONSIDERED PART OF YOUR PUBLICATION (IF ACCEPTED).
Please fill in these questions diligently. Information will not be copyedited, so please use proper spelling and grammar, use correct capitalization, and avoid abbreviations.

DO NOT FORGET TO SAVE AS PDF _AND_ CLICK THE SUBMIT BUTTON SO YOUR ANSWERS ARE IN OUR DATABASE !!!

Citation Suggestion (if you append the pdf as Appendix we suggest to cite this paper in the caption):
Eysenbach G, CONSORT-EHEALTH Group
CONSORT-EHEALTH: Improving and Standardizing Evaluation Reports of Web-based and Mobile Health Interventions
J Med Internet Res 2011;13(4):e126
URL: http://www.jmir.org/2011/4/e126/
doi: 10.2196/jmir.1923
PMID: 22209829

*Vereist

Your name *

First Last

Huis in het Veld

Primary Affiliation (short), City, Country *

University of Toronto, Toronto, Canada

Amsterdam UMC, Vrije Universiteit Amsterc

Your e-mail address *

abc@gmail.com

jghuisinhetveld@gmail.com

Title of your manuscript *

Provide the (draft) title of your manuscript.

Online Self-Management Support for Family Caregivers Dealing With Behavior Changes in Relatives With Dementia (Part 2): Randomized Controlled Trial

Name of your App/Software/Intervention *

If there is a short and a long/alternate name, write the short name first and add the long name in brackets.

Online Self-Management Support

Evaluated Version (if any)

e.g. "V1", "Release 2017-03-01", "Version 2.0.27913"

Jouw antwoord

Language(s) *

What language is the intervention/app in? If multiple languages are available, separate by comma (e.g. "English, French")

Dutch

URL of your Intervention Website or App

e.g. a direct link to the mobile app on app in appstore (itunes, Google Play), or URL of the website. If the intervention is a DVD or hardware, you can also link to an Amazon page.

https://www.nivel.nl/sites/default/files/bes

URL of an image/screenshot (optional)

Jouw antwoord

Accessibility *

Can an enduser access the intervention presently?

- (selected) access is free and open
- access only for special usergroups, not open
- access is open to everyone, but requires payment/subscription/in-app purchases
- app/intervention no longer accessible
- Anders:

Primary Medical Indication/Disease/Condition *

e.g. "Stress", "Diabetes", or define the target group in brackets after the condition, e.g. "Autism (Parents of children with)", "Alzheimers (Informal Caregivers of)"

"Dementia (informal Caregivers of)"

Primary Outcomes measured in trial *

comma-separated list of primary outcomes reported in the trial

"family caregivers' self-efficacy in dealing w

Secondary/other outcomes

Are there any other outcomes the intervention is expected to affect?

"Secondary outcomes were family caregivers' reports of behavior problems in the people with dementia and the quality of the relationship between the family caregiver and the person with dementia."

Recommended "Dose" *

What do the instructions for users say on how often the app should be used?

- ○ Approximately Daily
- ○ Approximately Weekly
- ○ Approximately Monthly
- ○ Approximately Yearly
- ◉ "as needed"
- ○ Anders:

Approx. Percentage of Users (starters) still using the app as recommended after 3 months *

- (x) unknown / not evaluated
- ( ) 0-10%
- ( ) 11-20%
- ( ) 21-30%
- ( ) 31-40%
- ( ) 41-50%
- ( ) 51-60%
- ( ) 61-70%
- ( ) 71%-80%
- ( ) 81-90%
- ( ) 91-100%
- ( ) Anders:

Overall, was the app/intervention effective? *

- ( ) yes: all primary outcomes were significantly better in intervention group vs control
- ( ) partly: SOME primary outcomes were significantly better in intervention group vs control
- ( ) no statistically significant difference between control and intervention
- ( ) potentially harmful: control was significantly better than intervention in one or more outcomes
- (x) inconclusive: more research is needed
- ( ) Anders:

Article Preparation Status/Stage *

At which stage in your article preparation are you currently (at the time you fill in this form)

- ( ) not submitted yet - in early draft status
- ( ) not submitted yet - in late draft status, just before submission
- ( ) submitted to a journal but not reviewed yet
- ( ) submitted to a journal and after receiving initial reviewer comments
- (x) submitted to a journal and accepted, but not published yet
- ( ) published
- ( ) Anders:

Journal *

If you already know where you will submit this paper (or if it is already submitted), please provide the journal name (if it is not JMIR, provide the journal name under "other")

- ○ not submitted yet / unclear where I will submit this
- ◉ Journal of Medical Internet Research (JMIR)
- ○ JMIR mHealth and UHealth
- ○ JMIR Serious Games
- ○ JMIR Mental Health
- ○ JMIR Public Health
- ○ JMIR Formative Research
- ○ Other JMIR sister journal
- ○ Anders:

Is this a full powered effectiveness trial or a pilot/feasibility trial? *

- ○ Pilot/feasibility
- ◉ Fully powered

Manuscript tracking number *

If this is a JMIR submission, please provide the manuscript tracking number under "other" (The ms tracking number can be found in the submission acknowledgement email, or when you login as author in JMIR. If the paper is already published in JMIR, then the ms tracking number is the four-digit number at the end of the DOI, to be found at the bottom of each published article in JMIR)

- ○ no ms number (yet) / not (yet) submitted to / published in JMIR
- ● Anders: #13001

TITLE AND ABSTRACT

1a) TITLE: Identification as a randomized trial in the title

1a) Does your paper address CONSORT item 1a? *

I.e does the title contain the phrase "Randomized Controlled Trial"? (if not, explain the reason under "other")

- ● yes
- ○ Anders:

1a-i) Identify the mode of delivery in the title

Identify the mode of delivery. Preferably use “web-based” and/or “mobile” and/or “electronic game” in the title. Avoid ambiguous terms like “online”, “virtual”, “interactive”. Use “Internet-based” only if Intervention includes non-web-based Internet components (e.g. email), use “computer-based” or “electronic” only if offline products are used. Use “virtual” only in the context of “virtual reality” (3-D worlds). Use “online” only in the context of “online support groups”. Complement or substitute product names with broader terms for the class of products (such as “mobile” or “smart phone” instead of “iphone”), especially if the application runs on different platforms.

	1	2	3	4	5	
subitem not at all important	○	○	◉	○	○	essential

Does your paper address subitem 1a-i? *

Copy and paste relevant sections from manuscript title (include quotes in quotation marks "like this" to indicate direct quotes from your manuscript), or elaborate on this item by providing additional information not in the ms, or briefly explain why the item is not applicable/relevant for your study

"Online Self-Management Support for Family Caregivers Dealing With Behavior Changes in Relatives With Dementia (Part 2): Randomized Controlled Trial"

1a-ii) Non-web-based components or important co-interventions in title

Mention non-web-based components or important co-interventions in title, if any (e.g., “with telephone support”).

	1	2	3	4	5	
subitem not at all important	○	○	◉	○	○	essential

Does your paper address subitem 1a-ii?

Copy and paste relevant sections from manuscript title (include quotes in quotation marks "like this" to indicate direct quotes from your manuscript), or elaborate on this item by providing additional information not in the ms, or briefly explain why the item is not applicable/relevant for your study

"Online Self-Management Support for Family Caregivers Dealing With Behavior Changes in Relatives With Dementia (Part 2): Randomized Controlled Trial"

1a-iii) Primary condition or target group in the title

Mention primary condition or target group in the title, if any (e.g., “for children with Type I Diabetes”) Example: A Web-based and Mobile Intervention with Telephone Support for Children with Type I Diabetes: Randomized Controlled Trial

	1	2	3	4	5	
subitem not at all important	○	○	○	●	○	essential

Does your paper address subitem 1a-iii? *

Copy and paste relevant sections from manuscript title (include quotes in quotation marks "like this" to indicate direct quotes from your manuscript), or elaborate on this item by providing additional information not in the ms, or briefly explain why the item is not applicable/relevant for your study

"Online Self-Management Support for Family Caregivers Dealing With Behavior Changes in Relatives With Dementia (Part 2): Randomized Controlled Trial"

1b) ABSTRACT: Structured summary of trial design, methods, results, and conclusions

NPT extension: Description of experimental treatment, comparator, care providers, centers, and blinding status.

1b-i) Key features/functionalities/components of the intervention and comparator in the METHODS section of the ABSTRACT

Mention key features/functionalities/components of the intervention and comparator in the abstract. If possible, also mention theories and principles used for designing the site. Keep in mind the needs of systematic reviewers and indexers by including important synonyms. (Note: Only report in the abstract what the main paper is reporting. If this information is missing from the main body of text, consider adding it)

	1	2	3	4	5	
subitem not at all important	○	○	○	◉	○	essential

Does your paper address subitem 1b-i? *

Copy and paste relevant sections from the manuscript abstract (include quotes in quotation marks "like this" to indicate direct quotes from your manuscript), or elaborate on this item by providing additional information not in the ms, or briefly explain why the item is not applicable/relevant for your study

"Participants were randomly assigned to one of (1) a major self-management support intervention consisting of personal e-mail contacts with a specialist dementia nurse, online videos, and e-bulletins; or (2) a medium intervention consisting only of online videos and e-bulletins; or (3) a minor intervention consisting of only the e-bulletins."

1b-ii) Level of human involvement in the METHODS section of the ABSTRACT

Clarify the level of human involvement in the abstract, e.g., use phrases like “fully automated” vs. “therapist/nurse/care provider/physician-assisted” (mention number and expertise of providers involved, if any). (Note: Only report in the abstract what the main paper is reporting. If this information is missing from the main body of text, consider adding it)

	1	2	3	4	5	
subitem not at all important	○	○	○	◉	○	essential

Does your paper address subitem 1b-ii?

Copy and paste relevant sections from the manuscript abstract (include quotes in quotation marks "like this" to indicate direct quotes from your manuscript), or elaborate on this item by providing additional information not in the ms, or briefly explain why the item is not applicable/relevant for your study

"The intervention - involving among others personal e-mail contacts with a dementia nurse - was compared to online interventions without these e-mail contacts."

1b-iii) Open vs. closed, web-based (self-assessment) vs. face-to-face assessments in the METHODS section of the ABSTRACT

Mention how participants were recruited (online vs. offline), e.g., from an open access website or from a clinic or a closed online user group (closed usergroup trial), and clarify if this was a purely web-based trial, or there were face-to-face components (as part of the intervention or for assessment). Clearly say if outcomes were self-assessed through questionnaires (as common in web-based trials). Note: In traditional offline trials, an open trial (open-label trial) is a type of clinical trial in which both the researchers and participants know which treatment is being administered. To avoid confusion, use “blinded” or “unblinded” to indicated the level of blinding instead of “open”, as “open” in web-based trials usually refers to “open access” (i.e. participants can self-enrol). (Note: Only report in the abstract what the main paper is reporting. If this information is missing from the main body of text, consider adding it)

	1	2	3	4	5	
subitem not at all important	○	●	○	○	○	essential

Does your paper address subitem 1b-iii?

Copy and paste relevant sections from the manuscript abstract (include quotes in quotation marks "like this" to indicate direct quotes from your manuscript), or elaborate on this item by providing additional information not in the ms, or briefly explain why the item is not applicable/relevant for your study

"The primary outcome was family caregivers’ self-efficacy in dealing with behavior changes of the relative with dementia. Secondary outcomes were family caregivers’ reports of behavior problems in the people with dementia and the quality of the relationship between the family caregiver and the person with dementia. Measurements were performed at the baseline and at six (T1) and twelve weeks

1b-iv) RESULTS section in abstract must contain use data

Report number of participants enrolled/assessed in each group, the use/uptake of the intervention (e.g., attrition/adherence metrics, use over time, number of logins etc.), in addition to primary/secondary outcomes. (Note: Only report in the abstract what the main paper is reporting. If this information is missing from the main body of text, consider adding it)

	1	2	3	4	5	
subitem not at all important	○	○	○	◉	○	essential

Does your paper address subitem 1b-iv?

Copy and paste relevant sections from the manuscript abstract (include quotes in quotation marks "like this" to indicate direct quotes from your manuscript), or elaborate on this item by providing additional information not in the ms, or briefly explain why the item is not applicable/relevant for your study

"A randomized controlled trial (RCT) was conducted with 81 family caregivers of people with dementia who live at home. Participants were randomly assigned to one of (1) a major self-management support intervention consisting of personal e-mail contacts with a specialist dementia nurse, online videos, and e-bulletins; or (2) a medium intervention consisting only of online videos and e-bulletins; or (3) a minor intervention consisting of only the e-bulletins."

1b-v) CONCLUSIONS/DISCUSSION in abstract for negative trials

Conclusions/Discussions in abstract for negative trials: Discuss the primary outcome - if the trial is negative (primary outcome not changed), and the intervention was not used, discuss whether negative results are attributable to lack of uptake and discuss reasons. (Note: Only report in the abstract what the main paper is reporting. If this information is missing from the main body of text, consider adding it)

	1	2	3	4	5	
subitem not at all important	○	○	○	◉	○	essential

Does your paper address subitem 1b-v?

Copy and paste relevant sections from the manuscript abstract (include quotes in quotation marks "like this" to indicate direct quotes from your manuscript), or elaborate on this item by providing additional information not in the ms, or briefly explain why the item is not applicable/relevant for your study

"The expectation that an online self-management support intervention involving e-mail contacts would lead to positive effects and be more effective than online interventions without personal e-mail contacts was not borne out. One explanation might be related to the fact that not all family caregivers who were assigned to that intervention actually made use of the opportunity for personal e-mail contact. The online videos were also not always viewed."

INTRODUCTION

2a) In INTRODUCTION: Scientific background and explanation of rationale

2a-i) Problem and the type of system/solution

Describe the problem and the type of system/solution that is object of the study: intended as stand-alone intervention vs. incorporated in broader health care program? Intended for a particular patient population? Goals of the intervention, e.g., being more cost-effective to other interventions, replace or complement other solutions? (Note: Details about the intervention are provided in “Methods” under 5)

	1	2	3	4	5	
subitem not at all important	○	○	●	○	○	essential

Does your paper address subitem 2a-i? *

Copy and paste relevant sections from the manuscript (include quotes in quotation marks "like this" to indicate direct quotes from your manuscript), or elaborate on this item by providing additional information not in the ms, or briefly explain why the item is not applicable/relevant for your study

"Although studies including online professional support have been developed and evaluated, most of them are aimed at general caregiving issues [15-18] and their overall quality of evidence is low [13]. Further research is required to clarify the necessity of personal contacts with a professional [17] when coping with behavior changes in their relative with dementia.
The aim of this study is therefore to assess (1) if a major multicomponent intervention, consisting of e-mail contacts with a specialized dementia nurse, videos, and e-bulletins is more effective than interventions without personal contacts and (2) if a medium intervention including videos and e-bulletins is more effective than a minor intervention including e-bulletins only."

2a-ii) Scientific background, rationale: What is known about the (type of) system

Scientific background, rationale: What is known about the (type of) system that is the object of the study (be sure to discuss the use of similar systems for other conditions/diagnoses, if appropiate), motivation for the study, i.e. what are the reasons for and what is the context for this specific study, from which stakeholder viewpoint is the study performed, potential impact of findings [2]. Briefly justify the choice of the comparator.

	1	2	3	4	5	
subitem not at all important	○	○	◉	○	○	essential

Does your paper address subitem 2a-ii? *

Copy and paste relevant sections from the manuscript (include quotes in quotation marks "like this" to indicate direct quotes from your manuscript), or elaborate on this item by providing additional information not in the ms, or briefly explain why the item is not applicable/relevant for your study

"Available systematic reviews suggest that online support might have positive effects for family caregivers, e.g. on their self-efficacy and other psychological or psychosocial outcomes [9-12].
Family caregivers could benefit from multicomponent online interventions combining information and tailored caregiving strategies [10]. In particular, family caregivers might benefit from additional personal online contact with health professionals [10, 13] as health professionals can help them apply generic information to their specific situation [14] and give tailored advice based on their needs."

2b) In INTRODUCTION: Specific objectives or hypotheses

Does your paper address CONSORT subitem 2b? *

Copy and paste relevant sections from the manuscript (include quotes in quotation marks "like this" to indicate direct quotes from your manuscript), or elaborate on this item by providing additional information not in the ms, or briefly explain why the item is not applicable/relevant for your study

In method section: "In this study, we expected that (1) both the major and medium intervention arms would lead to a greater improvement in self-efficacy than the minor intervention arm and that (2) the major intervention arm would show larger improvements in self-efficacy than the medium intervention arm."

METHODS

3a) Description of trial design (such as parallel, factorial) including allocation ratio

Does your paper address CONSORT subitem 3a? *

Copy and paste relevant sections from the manuscript (include quotes in quotation marks "like this" to indicate direct quotes from your manuscript), or elaborate on this item by providing additional information not in the ms, or briefly explain why the item is not applicable/relevant for your study

In method see "Design, Intervention Arms, and Elements" and "Inclusion and Randomization".

3b) Important changes to methods after trial commencement (such as eligibility criteria), with reasons

Does your paper address CONSORT subitem 3b? *

Copy and paste relevant sections from the manuscript (include quotes in quotation marks "like this" to indicate direct quotes from your manuscript), or elaborate on this item by providing additional information not in the ms, or briefly explain why the item is not applicable/relevant for your study

No changes were made after trial commencement.

3b-i) Bug fixes, Downtimes, Content Changes

Bug fixes, Downtimes, Content Changes: ehealth systems are often dynamic systems. A description of changes to methods therefore also includes important changes made on the intervention or comparator during the trial (e.g., major bug fixes or changes in the functionality or content) (5-iii) and other “unexpected events” that may have influenced study design such as staff changes, system failures/downtimes, etc. [2].

	1	2	3	4	5	
subitem not at all important	○	◉	○	○	○	essential

Does your paper address subitem 3b-i?

Copy and paste relevant sections from the manuscript (include quotes in quotation marks "like this" to indicate direct quotes from your manuscript), or elaborate on this item by providing additional information not in the ms, or briefly explain why the item is not applicable/relevant for your study

Not applicable.

4a) Eligibility criteria for participants

Does your paper address CONSORT subitem 4a? *

Copy and paste relevant sections from the manuscript (include quotes in quotation marks "like this" to indicate direct quotes from your manuscript), or elaborate on this item by providing additional information not in the ms, or briefly explain why the item is not applicable/relevant for your study

"Family caregivers were eligible to participate in the study if they were at least 18 years old, were a partner or relative of a person diagnosed with dementia who lives at home, had at least once-weekly contact with the person with dementia, had access to the Internet and gave online consent."

4a-i) Computer / Internet literacy

Computer / Internet literacy is often an implicit "de facto" eligibility criterion - this should be explicitly clarified.

	1	2	3	4	5	
subitem not at all important	○	◉	○	○	○	essential

Does your paper address subitem 4a-i?

Copy and paste relevant sections from the manuscript (include quotes in quotation marks "like this" to indicate direct quotes from your manuscript), or elaborate on this item by providing additional information not in the ms, or briefly explain why the item is not applicable/relevant for your study

"Family caregivers were eligible to participate in the study if they were at least 18 years old, were a partner or relative of a person diagnosed with dementia who lives at home, had at least once-weekly contact with the person with dementia, had access to the Internet and gave online consent."

4a-ii) Open vs. closed, web-based vs. face-to-face assessments:

Open vs. closed, web-based vs. face-to-face assessments: Mention how participants were recruited (online vs. offline), e.g., from an open access website or from a clinic, and clarify if this was a purely web-based trial, or there were face-to-face components (as part of the intervention or for assessment), i.e., to what degree got the study team to know the participant. In online-only trials, clarify if participants were quasi-anonymous and whether having multiple identities was possible or whether technical or logistical measures (e.g., cookies, email confirmation, phone calls) were used to detect/prevent these.

	1	2	3	4	5	
subitem not at all important	○	○	○	○	◉	essential

Does your paper address subitem 4a-ii? *

Copy and paste relevant sections from the manuscript (include quotes in quotation marks "like this" to indicate direct quotes from your manuscript), or elaborate on this item by providing additional information not in the ms, or briefly explain why the item is not applicable/relevant for your study

"Family caregivers were recruited via the Dutch Alzheimer Society's panel, the Dutch Alzheimer Society's online forum (with 7000 monthly visitors), the Dementie.nl website (https://dementie.nl), and the Dutch Alzheimer Society's social media accounts (Twitter and Facebook). Details of the recruitment procedure have been

4a-iii) Information giving during recruitment

Information given during recruitment. Specify how participants were briefed for recruitment and in the informed consent procedures (e.g., publish the informed consent documentation as appendix, see also item X26), as this information may have an effect on user self-selection, user expectation and may also bias results.

	1	2	3	4	5	
subitem not at all important	○	○	○	◉	○	essential

Does your paper address subitem 4a-iii?

Copy and paste relevant sections from the manuscript (include quotes in quotation marks "like this" to indicate direct quotes from your manuscript), or elaborate on this item by providing additional information not in the ms, or briefly explain why the item is not applicable/relevant for your study

"After online consent was given (see the study protocol for more detail [19]), family caregivers were randomly allocated by the researcher (JGH) to one of the three intervention arms using a randomization schedule."

4b) Settings and locations where the data were collected

Does your paper address CONSORT subitem 4b? *

Copy and paste relevant sections from the manuscript (include quotes in quotation marks "like this" to indicate direct quotes from your manuscript), or elaborate on this item by providing additional information not in the ms, or briefly explain why the item is not applicable/relevant for your study

"Only the research team members had access to the data. Agreements about how to archive, share, and store data were signed by the organizations responsible for collecting the data."

4b-i) Report if outcomes were (self-)assessed through online questionnaires

Clearly report if outcomes were (self-)assessed through online questionnaires (as common in web-based trials) or otherwise.

	1	2	3	4	5	
subitem not at all important	○	○	○	●	○	essential

Does your paper address subitem 4b-i? *

Copy and paste relevant sections from the manuscript (include quotes in quotation marks "like this" to indicate direct quotes from your manuscript), or elaborate on this item by providing additional information not in the ms, or briefly explain why the item is not applicable/relevant for your study

"The primary outcome variable (self-efficacy) was measured using the 'Trust in Own Abilities' (TRUST) instrument, a questionnaire in Dutch.";
"The first secondary outcome variable was the presence and reaction scores for mood and behavior problems, measured using the Dutch version of the Revised Memory and Behavioral Problem Checklist (RMBPC) [25, 26]. The RMBPC is a self-assessment questionnaire which can be broken down into scales for disruptive behavior (8 items), depression (9 items), and memory-related problems (7 items).";
"A second secondary outcome variable concerned the positive and negative aspects of the relationship between the person with dementia and the family caregiver. This was measured by the Dyadic Relationship Scale (DRS). The family caregiver version has 11 items in two subscales: dyadic strain (5 items) and positive dyadic interaction (6 items). Family caregivers were asked to rate the separate items on a 4-point scale (1 = 'strongly disagree', 2 = 'disagree', 3 = 'agree', 4 = 'strongly agree'). "

4b-ii) Report how institutional affiliations are displayed

Report how institutional affiliations are displayed to potential participants [on ehealth media], as affiliations with prestigious hospitals or universities may affect volunteer rates, use, and reactions with regards to an intervention.(Not a required item – describe only if this may bias results)

	1	2	3	4	5	
subitem not at all important	○	◉	○	○	○	essential

Does your paper address subitem 4b-ii?

Copy and paste relevant sections from the manuscript (include quotes in quotation marks "like this" to indicate direct quotes from your manuscript), or elaborate on this item by providing additional information not in the ms, or briefly explain why the item is not applicable/relevant for your study

Not applicable.

5) The interventions for each group with sufficient details to allow replication, including how and when they were actually administered

5-i) Mention names, credential, affiliations of the developers, sponsors, and owners

Mention names, credential, affiliations of the developers, sponsors, and owners [6] (if authors/evaluators are owners or developer of the software, this needs to be declared in a “Conflict of interest” section or mentioned elsewhere in the manuscript).

	1	2	3	4	5	
subitem not at all important	○	◉	○	○	○	essential

Does your paper address subitem 5-i?

Copy and paste relevant sections from the manuscript (include quotes in quotation marks "like this" to indicate direct quotes from your manuscript), or elaborate on this item by providing additional information not in the ms, or briefly explain why the item is not applicable/relevant for your study

Not applicable.

5-ii) Describe the history/development process

Describe the history/development process of the application and previous formative evaluations (e.g., focus groups, usability testing), as these will have an impact on adoption/use rates and help with interpreting results.

	1	2	3	4	5	
subitem not at all important	○	○	●	○	○	essential

Does your paper address subitem 5-ii?

Copy and paste relevant sections from the manuscript (include quotes in quotation marks "like this" to indicate direct quotes from your manuscript), or elaborate on this item by providing additional information not in the ms, or briefly explain why the item is not applicable/relevant for your study

The development process is described in Huis In Het Veld JG, Willemse BM, van Asch IF, Groot Zwaaftink RB, Verkade PJ, Veldhuijzen NJ, Verkaik R, Blom MM, Francke AL. Online Self-Management Support for Family Caregivers to Help Them Manage Behavior Changes in Their Relative With Dementia: Study Protocol for a Randomized Controlled Trial and a Process Evaluation. JMIR Res Protoc, 2017. 6(11): p. e239.

5-iii) Revisions and updating

Revisions and updating. Clearly mention the date and/or version number of the application/intervention (and comparator, if applicable) evaluated, or describe whether the intervention underwent major changes during the evaluation process, or whether the development and/or content was "frozen" during the trial. Describe dynamic components such as news feeds or changing content which may have an impact on the replicability of the intervention (for unexpected events see item 3b).

	1	2	3	4	5	
subitem not at all important	○	◉	○	○	○	essential

Does your paper address subitem 5-iii?

Copy and paste relevant sections from the manuscript (include quotes in quotation marks "like this" to indicate direct quotes from your manuscript), or elaborate on this item by providing additional information not in the ms, or briefly explain why the item is not applicable/relevant for your study

Not applicable.

5-iv) Quality assurance methods

Provide information on quality assurance methods to ensure accuracy and quality of information provided [1], if applicable.

	1	2	3	4	5	
subitem not at all important	○	◉	○	○	○	essential

Does your paper address subitem 5-iv?

Copy and paste relevant sections from the manuscript (include quotes in quotation marks "like this" to indicate direct quotes from your manuscript), or elaborate on this item by providing additional information not in the ms, or briefly explain why the item is not applicable/relevant for your study

Not applicable.

5-v) Ensure replicability by publishing the source code, and/or providing screenshots/screen-capture video, and/or providing flowcharts of the algorithms used

Ensure replicability by publishing the source code, and/or providing screenshots/screen-capture video, and/or providing flowcharts of the algorithms used. Replicability (i.e., other researchers should in principle be able to replicate the study) is a hallmark of scientific reporting.

	1	2	3	4	5	
subitem not at all important	○	●	○	○	○	essential

Does your paper address subitem 5-v?

Copy and paste relevant sections from the manuscript (include quotes in quotation marks "like this" to indicate direct quotes from your manuscript), or elaborate on this item by providing additional information not in the ms, or briefly explain why the item is not applicable/relevant for your study

The full intervention protocol is available in https://www.nivel.nl/sites/default/files/bestanden/Intervention-procotol-self-management-support-dementia.pdf.

5-vi) Digital preservation

Digital preservation: Provide the URL of the application, but as the intervention is likely to change or disappear over the course of the years; also make sure the intervention is archived (Internet Archive, webcitation.org, and/or publishing the source code or screenshots/videos alongside the article). As pages behind login screens cannot be archived, consider creating demo pages which are accessible without login.

	1	2	3	4	5	
subitem not at all important	○	○	◉	○	○	essential

Does your paper address subitem 5-vi?

Copy and paste relevant sections from the manuscript (include quotes in quotation marks "like this" to indicate direct quotes from your manuscript), or elaborate on this item by providing additional information not in the ms, or briefly explain why the item is not applicable/relevant for your study

"For more details, the readers are referred to the full intervention protocol, available in https://www.nivel.nl/sites/default/files/bestanden/Intervention-procotol-self-management-support-dementia.pdf."

5-vii) Access

Access: Describe how participants accessed the application, in what setting/context, if they had to pay (or were paid) or not, whether they had to be a member of specific group. If known, describe how participants obtained “access to the platform and Internet” [1]. To ensure access for editors/reviewers/readers, consider to provide a “backdoor” login account or demo mode for reviewers/readers to explore the application (also important for archiving purposes, see vi).

	1	2	3	4	5	
subitem not at all important	○	○	◉	○	○	essential

Does your paper address subitem 5-vii? *

Copy and paste relevant sections from the manuscript (include quotes in quotation marks "like this" to indicate direct quotes from your manuscript), or elaborate on this item by providing additional information not in the ms, or briefly explain why the item is not applicable/relevant for your study

Is described elsewhere (in Huis In Het Veld JG, Willemse BM, van Asch IF, Groot Zwaaftink RB, Verkade PJ, Veldhuijzen NJ, Verkaik R, Blom MM, Francke AL. Online Self-Management Support for Family Caregivers to Help Them Manage Behavior Changes in Their Relative With Dementia: Study Protocol for a Randomized Controlled Trial and a Process Evaluation. JMIR Res Protoc, 2017. 6(11): p. e239).

5-viii) Mode of delivery, features/functionalities/components of the intervention and comparator, and the theoretical framework

Describe mode of delivery, features/functionalities/components of the intervention and comparator, and the theoretical framework [6] used to design them (instructional strategy [1], behaviour change techniques, persuasive features, etc., see e.g., [7, 8] for terminology). This includes an in-depth description of the content (including where it is coming from and who developed it) [1],” whether [and how] it is tailored to individual circumstances and allows users to track their progress and receive feedback” [6]. This also includes a description of communication delivery channels and – if computer-mediated communication is a component – whether communication was synchronous or asynchronous [6]. It also includes information on presentation strategies [1], including page design principles, average amount of text on pages, presence of hyperlinks to other resources, etc. [1].

	1	2	3	4	5	
subitem not at all important	○	○	◉	○	○	essential

Does your paper address subitem 5-viii? *

Copy and paste relevant sections from the manuscript (include quotes in quotation marks "like this" to indicate direct quotes from your manuscript), or elaborate on this item by providing additional information not in the ms, or briefly explain why the item is not applicable/relevant for your study

"The intervention arms are described elsewhere in more detail [19]. In short, the major intervention arm consisted of
(a) three personal e-mail contacts with a specialist dementia nurse (in a period of 12 weeks). The nurse supported the family caregivers in managing behavior changes by giving feedback on assignments (see element b) and tailoring support to the personal needs and questions of the family caregivers. Nurses were trained in a one-day course in which the intervention was further explained by the two researchers (JGH and IA). A peer review session, in which all nurses who provided the intervention were involved, took place halfway through the study period. In this peer review session, the nurses reflected together on the online support that they had given
(b) Six online videos about different types of behavior changes. Family caregivers could choose how many videos they watched and the assignments that they did themselves.
(c) Six e-bulletins containing practical information about different types of changes in behavior and how to manage them.
The medium intervention arm consisted only of the online videos and e-bulletins (see elements b and c above) and the minor intervention arm consisted only of the e-bulletins (see element c). For more details, the readers are referred to the full

5-ix) Describe use parameters

Describe use parameters (e.g., intended "doses" and optimal timing for use). Clarify what instructions or recommendations were given to the user, e.g., regarding timing, frequency, heaviness of use, if any, or was the intervention used ad libitum.

	1	2	3	4	5	
subitem not at all important	○	○	◉	○	○	essential

Does your paper address subitem 5-ix?

Copy and paste relevant sections from the manuscript (include quotes in quotation marks "like this" to indicate direct quotes from your manuscript), or elaborate on this item by providing additional information not in the ms, or briefly explain why the item is not applicable/relevant for your study

Is described elsewhere (in Huis In Het Veld JG, Willemse BM, van Asch IF, Groot Zwaaftink RB, Verkade PJ, Veldhuijzen NJ, Verkaik R, Blom MM, Francke AL. Online Self-Management Support for Family Caregivers to Help Them Manage Behavior Changes in Their Relative With Dementia: Study Protocol for a Randomized Controlled Trial and a Process Evaluation. JMIR Res Protoc, 2017. 6(11): p. e239).

5-x) Clarify the level of human involvement

Clarify the level of human involvement (care providers or health professionals, also technical assistance) in the e-intervention or as co-intervention (detail number and expertise of professionals involved, if any, as well as “type of assistance offered, the timing and frequency of the support, how it is initiated, and the medium by which the assistance is delivered”. It may be necessary to distinguish between the level of human involvement required for the trial, and the level of human involvement required for a routine application outside of a RCT setting (discuss under item 21 – generalizability).

	1	2	3	4	5	
subitem not at all important	○	○	○	◉	○	essential

Does your paper address subitem 5-x?

Copy and paste relevant sections from the manuscript (include quotes in quotation marks "like this" to indicate direct quotes from your manuscript), or elaborate on this item by providing additional information not in the ms, or briefly explain why the item is not applicable/relevant for your study

"The intervention arms are described elsewhere in more detail [19]. In short, the major intervention arm consisted of
(a) three personal e-mail contacts with a specialist dementia nurse (in a period of 12 weeks). The nurse supported the family caregivers in managing behavior changes by giving feedback on assignments (see element b) and tailoring support to the personal needs and questions of the family caregivers. Nurses were trained in a one-day course in which the intervention was further explained by the two researchers (JGH and IA). A peer review session, in which all nurses who provided the intervention were involved, took place halfway through the study period. In this peer review session, the nurses reflected together on the online support that they had given
(b) Six online videos about different types of behavior changes. Family caregivers could choose how many videos they watched and the assignments that they did themselves.
(c) Six e-bulletins containing practical information about different types of changes in behavior and how to manage them.
The medium intervention arm consisted only of the online videos and e-bulletins (see elements b and c above) and the minor intervention arm consisted only of the e-bulletins (see element c). For more details, the readers are referred to the full

5-xi) Report any prompts/reminders used

Report any prompts/reminders used: Clarify if there were prompts (letters, emails, phone calls, SMS) to use the application, what triggered them, frequency etc. It may be necessary to distinguish between the level of prompts/reminders required for the trial, and the level of prompts/reminders for a routine application outside of a RCT setting (discuss under item 21 – generalizability).

	1	2	3	4	5	
subitem not at all important	○	○	○	◉	○	essential

Does your paper address subitem 5-xi? *

Copy and paste relevant sections from the manuscript (include quotes in quotation marks "like this" to indicate direct quotes from your manuscript), or elaborate on this item by providing additional information not in the ms, or briefly explain why the item is not applicable/relevant for your study

Is described elsewhere (in Huis In Het Veld JG, Willemse BM, van Asch IF, Groot Zwaaftink RB, Verkade PJ, Veldhuijzen NJ, Verkaik R, Blom MM, Francke AL. Online Self-Management Support for Family Caregivers to Help Them Manage Behavior Changes in Their Relative With Dementia: Study Protocol for a Randomized Controlled Trial and a Process Evaluation. JMIR Res Protoc, 2017. 6(11): p. e239).

5-xii) Describe any co-interventions (incl. training/support)

Describe any co-interventions (incl. training/support): Clearly state any interventions that are provided in addition to the targeted eHealth intervention, as ehealth intervention may not be designed as stand-alone intervention. This includes training sessions and support [1]. It may be necessary to distinguish between the level of training required for the trial, and the level of training for a routine application outside of a RCT setting (discuss under item 21 – generalizability.

	1	2	3	4	5	
subitem not at all important	○	○	○	◉	○	essential

Does your paper address subitem 5-xii? *

Copy and paste relevant sections from the manuscript (include quotes in quotation marks "like this" to indicate direct quotes from your manuscript), or elaborate on this item by providing additional information not in the ms, or briefly explain why the item is not applicable/relevant for your study

"The intervention arms are described elsewhere in more detail [19]. In short, the major intervention arm consisted of
(a) three personal e-mail contacts with a specialist dementia nurse (in a period of 12 weeks). The nurse supported the family caregivers in managing behavior changes by giving feedback on assignments (see element b) and tailoring support to the personal needs and questions of the family caregivers. Nurses were trained in a one-day course in which the intervention was further explained by the two researchers (JGH and IA). A peer review session, in which all nurses who provided the intervention were involved, took place halfway through the study period. In this peer review session, the nurses reflected together on the online support that they had given
(b) Six online videos about different types of behavior changes. Family caregivers could choose how many videos they watched and the assignments that they did themselves.
(c) Six e-bulletins containing practical information about different types of changes in behavior and how to manage them.
The medium intervention arm consisted only of the online videos and e-bulletins (see elements b and c above) and the minor intervention arm consisted only of the e-bulletins (see element c). For more details, the readers are referred to the full

6a) Completely defined pre-specified primary and secondary outcome measures, including how and when they were assessed

Does your paper address CONSORT subitem 6a? *

Copy and paste relevant sections from the manuscript (include quotes in quotation marks "like this" to indicate direct quotes from your manuscript), or elaborate on this item by providing additional information not in the ms, or briefly explain why the item is not applicable/relevant for your study

See in method section: "Measurement Procedures", "Primary Outcome" and "Secondary Outcomes".

6a-i) Online questionnaires: describe if they were validated for online use and apply CHERRIES items to describe how the questionnaires were designed/deployed

If outcomes were obtained through online questionnaires, describe if they were validated for online use and apply CHERRIES items to describe how the questionnaires were designed/deployed [9].

	1	2	3	4	5	
subitem not at all important	○	○	○	◉	○	essential

Does your paper address subitem 6a-i?

Copy and paste relevant sections from manuscript text

"The primary outcome variable (self-efficacy) was measured using the 'Trust in Own Abilities' (TRUST) instrument, a questionnaire in Dutch. The questionnaire had been used previously to measure self-efficacy in family caregivers of people with dementia living at home [23].";
"The RMBPC is a self-assessment questionnaire which can be broken down into scales for disruptive behavior (8 items), depression (9 items), and memory-related problems (7 items). Overall reliability for this scale is .84 for patient behavior and .90 for caregiver reaction [25].";
"A second secondary outcome variable concerned the positive and negative aspects of the relationship between the person with dementia and the family caregiver. This was measured by the Dyadic Relationship Scale (DRS). The family caregiver version has 11 items in two subscales: dyadic strain (5 items) and positive dyadic interaction (6 items). Family caregivers were asked to rate the separate items on a 4-point scale (1 = 'strongly disagree', 2 = 'disagree', 3 = 'agree', 4 = 'strongly agree'). Overall reliability for this scale is .89 for negative dyadic strain and .85 for positive dyadic interaction [28]."

6a-ii) Describe whether and how “use” (including intensity of use/dosage) was defined/measured/monitored

Describe whether and how “use” (including intensity of use/dosage) was defined/measured/monitored (logins, logfile analysis, etc.). Use/adoption metrics are important process outcomes that should be reported in any ehealth trial.

	1	2	3	4	5	
subitem not at all important	○	○	○	◉	○	essential

Does your paper address subitem 6a-ii?

Copy and paste relevant sections from manuscript text

Is described elsewhere (in Huis In Het Veld JG, Willemse BM, van Asch IF, Groot Zwaaftink RB, Verkade PJ, Veldhuijzen NJ, Verkaik R, Blom MM, Francke AL. Online Self-Management Support for Family Caregivers to Help Them Manage Behavior Changes in Their Relative With Dementia: Study Protocol for a Randomized Controlled Trial and a Process Evaluation. JMIR Res Protoc, 2017. 6(11): p. e239).
Results are reported in Huis In Het Veld JG, van Asch IFM, Willemse BM, Verkade PJ, Pot AM, Blom MM, Groot Zwaaftink RBM, Francke AL. Process Evaluation of Nurse-Led Online Self-Management Support for Family Caregivers to Deal With Behavior Changes of a Relative With Dementia (Part 1): Mixed Methods Study. J Med Internet Res. 2019 Oct 11;21(10):e13002. doi: 10.2196/13002.

6a-iii) Describe whether, how, and when qualitative feedback from participants was obtained

Describe whether, how, and when qualitative feedback from participants was obtained (e.g., through emails, feedback forms, interviews, focus groups).

	1	2	3	4	5	
subitem not at all important	○	○	○	◉	○	essential

Does your paper address subitem 6a-iii?

Copy and paste relevant sections from manuscript text

"Alongside the RCT, a mixed-method process evaluation was performed to evaluate the online self-management support intervention in terms of usability and

6b) Any changes to trial outcomes after the trial commenced, with reasons

Does your paper address CONSORT subitem 6b? *

Copy and paste relevant sections from the manuscript (include quotes in quotation marks "like this" to indicate direct quotes from your manuscript), or elaborate on this item by providing additional information not in the ms, or briefly explain why the item is not applicable/relevant for your study

Not applicable. No changes were made to trial outcomes.

7a) How sample size was determined

NPT: When applicable, details of whether and how the clustering by care provides or centers was addressed

7a-i) Describe whether and how expected attrition was taken into account when calculating the sample size

Describe whether and how expected attrition was taken into account when calculating the sample size.

	1	2	3	4	5	
subitem not at all important	○	○	○	○	●	essential

Does your paper address subitem 7a-i?

Copy and paste relevant sections from manuscript title (include quotes in quotation marks "like this" to indicate direct quotes from your manuscript), or elaborate on this item by providing additional information not in the ms, or briefly explain why the item is not applicable/relevant for your study

"Based on a difference of 0.8 standard deviation units between the groups and assuming a significance level of 5%, a power of 80%, and correlation of 0.6 between the two repeated measures, the number of subjects needed per group was 20. Taking into account a drop-out rate of 20%, 24 participants per group were needed."

7b) When applicable, explanation of any interim analyses and stopping guidelines

Does your paper address CONSORT subitem 7b? *

Copy and paste relevant sections from the manuscript (include quotes in quotation marks "like this" to indicate direct quotes from your manuscript), or elaborate on this item by providing additional information not in the ms, or briefly explain why the item is not applicable/relevant for your study

Not applicable.

8a) Method used to generate the random allocation sequence

NPT: When applicable, how care providers were allocated to each trial group

Does your paper address CONSORT subitem 8a? *

Copy and paste relevant sections from the manuscript (include quotes in quotation marks "like this" to indicate direct quotes from your manuscript), or elaborate on this item by providing additional information not in the ms, or briefly explain why the item is not applicable/relevant for your study

Not applicable.

8b) Type of randomisation; details of any restriction (such as blocking and block size)

Does your paper address CONSORT subitem 8b? *

Copy and paste relevant sections from the manuscript (include quotes in quotation marks "like this" to indicate direct quotes from your manuscript), or elaborate on this item by providing additional information not in the ms, or briefly explain why the item is not applicable/relevant for your study

"After online consent was given (see the study protocol for more detail [19]), family caregivers were randomly allocated by the researcher (JGH) to one of the three intervention arms using a randomization schedule. Block randomization was applied to achieve an equal likelihood of the participant being allocated to each of the three intervention arms [20]. An independent epidemiologist prepared a randomization schedule using several block sizes of 6 and 9."

9) Mechanism used to implement the random allocation sequence (such as sequentially numbered containers), describing any steps taken to conceal the sequence until interventions were assigned

Does your paper address CONSORT subitem 9? *

Copy and paste relevant sections from the manuscript (include quotes in quotation marks "like this" to indicate direct quotes from your manuscript), or elaborate on this item by providing additional information not in the ms, or briefly explain why the item is not applicable/relevant for your study

"After online consent was given (see the study protocol for more detail [19]), family caregivers were randomly allocated by the researcher (JGH) to one of the three intervention arms using a randomization schedule. Block randomization was applied to achieve an equal likelihood of the participant being allocated to each of the three intervention arms [20]. An independent epidemiologist prepared a randomization schedule using several block sizes of 6 and 9."

10) Who generated the random allocation sequence, who enrolled participants, and who assigned participants to interventions

Does your paper address CONSORT subitem 10? *

Copy and paste relevant sections from the manuscript (include quotes in quotation marks "like this" to indicate direct quotes from your manuscript), or elaborate on this item by providing additional information not in the ms, or briefly explain why the item is not applicable/relevant for your study

"After online consent was given (see the study protocol for more detail [19]), family caregivers were randomly allocated by the researcher (JGH) to one of the three intervention arms using a randomization schedule. Block randomization was applied to achieve an equal likelihood of the participant being allocated to each of the three intervention arms [20]. An independent epidemiologist prepared a randomization schedule using several block sizes of 6 and 9."

11a) If done, who was blinded after assignment to interventions (for example, participants, care providers, those assessing outcomes) and how

NPT: Whether or not administering co-interventions were blinded to group assignment

11a-i) Specify who was blinded, and who wasn't

Specify who was blinded, and who wasn't. Usually, in web-based trials it is not possible to blind the participants [1, 3] (this should be clearly acknowledged), but it may be possible to blind outcome assessors, those doing data analysis or those administering co-interventions (if any).

	1	2	3	4	5	
subitem not at all important	○	○	○	●	○	essential

Does your paper address subitem 11a-i? *

Copy and paste relevant sections from the manuscript (include quotes in quotation marks "like this" to indicate direct quotes from your manuscript), or elaborate on this item by providing additional information not in the ms, or briefly explain why the item is not applicable/relevant for your study

"Participants could not be blinded as it is impossible to blind participants to the sort of eHealth intervention they are receiving [21]."

11a-ii) Discuss e.g., whether participants knew which intervention was the “intervention of interest” and which one was the “comparator”

Informed consent procedures (4a-ii) can create biases and certain expectations - discuss e.g., whether participants knew which intervention was the “intervention of interest” and which one was the “comparator”.

	1	2	3	4	5	
subitem not at all important	○	○	○	●	○	essential

Does your paper address subitem 11a-ii?

Copy and paste relevant sections from the manuscript (include quotes in quotation marks "like this" to indicate direct quotes from your manuscript), or elaborate on this item by providing additional information not in the ms, or briefly explain why the item is not applicable/relevant for your study

Participants were informed about the three options. More detials are described elsewhere (in Huis In Het Veld JG, Willemse BM, van Asch IF, Groot Zwaaftink RB, Verkade PJ, Veldhuijzen NJ, Verkaik R, Blom MM, Francke AL. Online Self-Management Support for Family Caregivers to Help Them Manage Behavior Changes in Their Relative With Dementia: Study Protocol for a Randomized Controlled Trial and a Process Evaluation. JMIR Res Protoc, 2017. 6(11): p. e239).

11b) If relevant, description of the similarity of interventions

(this item is usually not relevant for ehealth trials as it refers to similarity of a placebo or sham intervention to a active medication/intervention)

Does your paper address CONSORT subitem 11b? *

Copy and paste relevant sections from the manuscript (include quotes in quotation marks "like this" to indicate direct quotes from your manuscript), or elaborate on this item by providing additional information not in the ms, or briefly explain why the item is not applicable/relevant for your study

"The intervention arms are described elsewhere in more detail [19]. In short, the major intervention arm consisted of
(a) three personal e-mail contacts with a specialist dementia nurse (in a period of 12 weeks). The nurse supported the family caregivers in managing behavior changes by giving feedback on assignments (see element b) and tailoring support to the personal needs and questions of the family caregivers. Nurses were trained in a one-day course in which the intervention was further explained by the two researchers (JGH and IA). A peer review session, in which all nurses who provided the intervention were involved, took place halfway through the study period. In this peer review session, the nurses reflected together on the online support that they had given
(b) Six online videos about different types of behavior changes. Family caregivers could choose how many videos they watched and the assignments that they did themselves.
(c) Six e-bulletins containing practical information about different types of changes in behavior and how to manage them.
The medium intervention arm consisted only of the online videos and e-bulletins (see elements b and c above) and the minor intervention arm consisted only of the e-bulletins (see element c). For more details, the readers are referred to the full

12a) Statistical methods used to compare groups for primary and secondary outcomes

NPT: When applicable, details of whether and how the clustering by care providers or centers was addressed

Does your paper address CONSORT subitem 12a? *

Copy and paste relevant sections from the manuscript (include quotes in quotation marks "like this" to indicate direct quotes from your manuscript), or elaborate on this item by providing additional information not in the ms, or briefly explain why the item is not applicable/relevant for your study

"All data were analyzed using SPSS Software (version 22.0). Mixed-model analyses were carried out to compare primary and secondary outcomes between the major and the minor intervention arm and between the medium and minor intervention arm over time and at T1 and T2. Mixed-model analyses were performed to take account of the correlation between the two repeated measurements within the subject (T1 and T2). To obtain the intervention effect at two different time points, time and interaction between intervention and time were added to the model. All mixed-model analyses were adjusted for the baseline value of the particular outcome. In addition to crude effects, effects adjusted for gender, type of relationship, appearance of first symptoms, education level and shared caregiving were also estimated."

12a-i) Imputation techniques to deal with attrition / missing values

Imputation techniques to deal with attrition / missing values: Not all participants will use the intervention/comparator as intended and attrition is typically high in ehealth trials. Specify how participants who did not use the application or dropped out from the trial were treated in the statistical analysis (a complete case analysis is strongly discouraged, and simple imputation techniques such as LOCF may also be problematic [4]).

	1	2	3	4	5	
subitem not at all important	○	○	●	○	○	essential

Does your paper address subitem 12a-i? *

Copy and paste relevant sections from the manuscript (include quotes in quotation marks "like this" to indicate direct quotes from your manuscript), or elaborate on this item by providing additional information not in the ms, or briefly explain why the item is not applicable/relevant for your study

"All data were analyzed using SPSS Software (version 22.0). Mixed-model analyses were carried out to compare primary and secondary outcomes between the major and the minor intervention arm and between the medium and minor intervention arm over time and at T1 and T2. Mixed-model analyses were performed to take account of the correlation between the two repeated measurements within the subject (T1 and T2). To obtain the intervention effect at two different time points, time and interaction between intervention and time were added to the model. All mixed-model analyses were adjusted for the baseline value of the particular outcome. In addition to crude effects, effects adjusted for gender, type of relationship, appearance of first symptoms, education level and shared caregiving were also estimated."

12b) Methods for additional analyses, such as subgroup analyses and adjusted analyses

Does your paper address CONSORT subitem 12b? *

Copy and paste relevant sections from the manuscript (include quotes in quotation marks "like this" to indicate direct quotes from your manuscript), or elaborate on this item by providing additional information not in the ms, or briefly explain why the item is not applicable/relevant for your study

"All data were analyzed using SPSS Software (version 22.0). Mixed-model analyses were carried out to compare primary and secondary outcomes between the major and the minor intervention arm and between the medium and minor intervention arm over time and at T1 and T2. Mixed-model analyses were performed to take account of the correlation between the two repeated measurements within the subject (T1 and T2). To obtain the intervention effect at two different time points, time and interaction between intervention and time were added to the model. All mixed-model analyses were adjusted for the baseline value of the particular outcome. In addition to crude effects, effects adjusted for gender, type of relationship, appearance of first symptoms, education level and shared caregiving were also estimated."

X26) REB/IRB Approval and Ethical Considerations [recommended as subheading under "Methods"] (not a CONSORT item)

X26-i) Comment on ethics committee approval

	1	2	3	4	5	
subitem not at all important	○	○	○	●	○	essential

Does your paper address subitem X26-i?

Copy and paste relevant sections from the manuscript (include quotes in quotation marks "like this" to indicate direct quotes from your manuscript), or elaborate on this item by providing additional information not in the ms, or briefly explain why the item is not applicable/relevant for your study

"The study was approved by the VU University Medical Center's Medical Ethics Committee (reference 2016.559). It had no objections to the study."

x26-ii) Outline informed consent procedures

Outline informed consent procedures e.g., if consent was obtained offline or online (how? Checkbox, etc.?), and what information was provided (see 4a-ii). See [6] for some items to be included in informed consent documents.

	1	2	3	4	5	
subitem not at all important	○	○	○	●	○	essential

Does your paper address subitem X26-ii?

Copy and paste relevant sections from the manuscript (include quotes in quotation marks "like this" to indicate direct quotes from your manuscript), or elaborate on this item by providing additional information not in the ms, or briefly explain why the item is not applicable/relevant for your study

"All participants were required to give their informed consent for participation via an online informed consent form."

X26-iii) Safety and security procedures

Safety and security procedures, incl. privacy considerations, and any steps taken to reduce the likelihood or detection of harm (e.g., education and training, availability of a hotline)

	1	2	3	4	5	
subitem not at all important	○	◉	○	○	○	essential

Does your paper address subitem X26-iii?

Copy and paste relevant sections from the manuscript (include quotes in quotation marks "like this" to indicate direct quotes from your manuscript), or elaborate on this item by providing additional information not in the ms, or briefly explain why the item is not applicable/relevant for your study

Not applicable.

RESULTS

13a) For each group, the numbers of participants who were randomly assigned, received intended treatment, and were analysed for the primary outcome

NPT: The number of care providers or centers performing the intervention in each group and the number of patients treated by each care provider in each center

Does your paper address CONSORT subitem 13a? *

Copy and paste relevant sections from the manuscript (include quotes in quotation marks "like this" to indicate direct quotes from your manuscript), or elaborate on this item by providing additional information not in the ms, or briefly explain why the item is not applicable/relevant for your study

"A total of 158 family caregivers expressed their interest in participating in the study. After sending an information letter, the first 81 caregivers who signed the online informed consent form and completed the baseline assessment were included.
After completing the baseline questionnaire, the participants were randomly allocated to the major (27), medium (27), or minor (27) intervention arms following the block randomization schedule. A total of 70 (86.4%) family caregivers completed the T1 assessment (6 weeks after baseline), and 66 (81.5%) family caregivers completed the T2 assessment (12 weeks after baseline) (see Table 1)."

13b) For each group, losses and exclusions after randomisation, together with reasons

Does your paper address CONSORT subitem 13b? (NOTE: Preferably, this is shown in a CONSORT flow diagram) *

Copy and paste relevant sections from the manuscript (include quotes in quotation marks "like this" to indicate direct quotes from your manuscript), or elaborate on this item by providing additional information not in the ms, or briefly explain why the item is not applicable/relevant for your study

This is shown in a CONSORT flow diagram, see table 1.

13b-i) Attrition diagram

Strongly recommended: An attrition diagram (e.g., proportion of participants still logging in or using the intervention/comparator in each group plotted over time, similar to a survival curve) or other figures or tables demonstrating usage/dose/engagement.

	1	2	3	4	5	
subitem not at all important	○	○	●	○	○	essential

Does your paper address subitem 13b-i?

Copy and paste relevant sections from the manuscript or cite the figure number if applicable (include quotes in quotation marks "like this" to indicate direct quotes from your manuscript), or elaborate on this item by providing additional information not in the ms, or briefly explain why the item is not applicable/relevant for your study

Not applicable.

14a) Dates defining the periods of recruitment and follow-up

Does your paper address CONSORT subitem 14a? *

Copy and paste relevant sections from the manuscript (include quotes in quotation marks "like this" to indicate direct quotes from your manuscript), or elaborate on this item by providing additional information not in the ms, or briefly explain why the item is not applicable/relevant for your study

"A three-arm randomized controlled trial (RCT) was carried out between March 2017 and August 2017 in the Netherlands."

14a-i) Indicate if critical “secular events” fell into the study period

Indicate if critical “secular events” fell into the study period, e.g., significant changes in Internet resources available or “changes in computer hardware or Internet delivery resources”

	1	2	3	4	5	
subitem not at all important	○	◉	○	○	○	essential

Does your paper address subitem 14a-i?

Copy and paste relevant sections from the manuscript (include quotes in quotation marks "like this" to indicate direct quotes from your manuscript), or elaborate on this item by providing additional information not in the ms, or briefly explain why the item is not applicable/relevant for your study

Not applicable. There were no "secular events" in the study period.

14b) Why the trial ended or was stopped (early)

Does your paper address CONSORT subitem 14b? *

Copy and paste relevant sections from the manuscript (include quotes in quotation marks "like this" to indicate direct quotes from your manuscript), or elaborate on this item by providing additional information not in the ms, or briefly explain why the item is not applicable/relevant for your study

"A total of 158 family caregivers expressed their interest in participating in the study. After sending an information letter, the first 81 caregivers who signed the online informed consent form and completed the baseline assessment were included."

15) A table showing baseline demographic and clinical characteristics for each group

NPT: When applicable, a description of care providers (case volume, qualification, expertise, etc.) and centers (volume) in each group

Does your paper address CONSORT subitem 15? *

Copy and paste relevant sections from the manuscript (include quotes in quotation marks "like this" to indicate direct quotes from your manuscript), or elaborate on this item by providing additional information not in the ms, or briefly explain why the item is not applicable/relevant for your study

See in Results section "table 2. Baseline data for the caregivers included".

15-i) Report demographics associated with digital divide issues

In ehealth trials it is particularly important to report demographics associated with digital divide issues, such as age, education, gender, social-economic status, computer/Internet/ehealth literacy of the participants, if known.

	1	2	3	4	5	
subitem not at all important	○	○	○	◉	○	essential

Does your paper address subitem 15-i? *

Copy and paste relevant sections from the manuscript (include quotes in quotation marks "like this" to indicate direct quotes from your manuscript), or elaborate on this item by providing additional information not in the ms, or briefly explain why the item is not applicable/relevant for your study

See in Results section "table 2. Baseline data for the caregivers included".

16) For each group, number of participants (denominator) included in each analysis and whether the analysis was by original assigned groups

16-i) Report multiple “denominators” and provide definitions

Report multiple “denominators” and provide definitions: Report N’s (and effect sizes) “across a range of study participation [and use] thresholds” [1], e.g., N exposed, N consented, N used more than x times, N used more than y weeks, N participants “used” the intervention/comparator at specific pre-defined time points of interest (in absolute and relative numbers per group). Always clearly define “use” of the intervention.

	1	2	3	4	5	
subitem not at all important	○	○	◉	○	○	essential

Does your paper address subitem 16-i? *

Copy and paste relevant sections from the manuscript (include quotes in quotation marks "like this" to indicate direct quotes from your manuscript), or elaborate on this item by providing additional information not in the ms, or briefly explain why the item is not applicable/relevant for your study

Results are reported in Huis In Het Veld JG, van Asch IFM, Willemse BM, Verkade PJ, Pot AM, Blom MM, Groot Zwaaftink RBM, Francke AL. Process Evaluation of Nurse-Led Online Self-Management Support for Family Caregivers to Deal With Behavior Changes of a Relative With Dementia (Part 1): Mixed Methods Study. J Med Internet Res. 2019 Oct 11;21(10):e13002. doi: 10.2196/13002.

16-ii) Primary analysis should be intent-to-treat

Primary analysis should be intent-to-treat, secondary analyses could include comparing only “users”, with the appropriate caveats that this is no longer a randomized sample (see 18-i).

	1	2	3	4	5	
subitem not at all important	○	○	◉	○	○	essential

Does your paper address subitem 16-ii?

Copy and paste relevant sections from the manuscript (include quotes in quotation marks "like this" to indicate direct quotes from your manuscript), or elaborate on this item by providing additional information not in the ms, or briefly explain why the item is not applicable/relevant for your study

In this trial it was difficult to define 'users' or 'non-users'. See our reflections on this in Huis In Het Veld JG, van Asch IFM, Willemse BM, Verkade PJ, Pot AM, Blom MM, Groot Zwaaftink RBM, Francke AL. Process Evaluation of Nurse-Led Online Self-Management Support for Family Caregivers to Deal With Behavior Changes of a Relative With Dementia (Part 1): Mixed Methods Study. J Med Internet Res. 2019 Oct 11;21(10):e13002. doi: 10.2196/13002.

17a) For each primary and secondary outcome, results for each group, and the estimated effect size and its precision (such as 95% confidence interval)

Does your paper address CONSORT subitem 17a? *

Copy and paste relevant sections from the manuscript (include quotes in quotation marks "like this" to indicate direct quotes from your manuscript), or elaborate on this item by providing additional information not in the ms, or briefly explain why the item is not applicable/relevant for your study

In method section see table 3 "Results for the major intervention arm compared to the minor intervention arm over time, at T1 and at T2 on the outcomes TRUST, RMBPC and DRS" and table 4 "Results for the medium intervention arm compared to the minor intervention arm over time, at T1 and at T2 on the outcomes TRUST, RMBPC and DRS".

17a-i) Presentation of process outcomes such as metrics of use and intensity of use

In addition to primary/secondary (clinical) outcomes, the presentation of process outcomes such as metrics of use and intensity of use (dose, exposure) and their operational definitions is critical. This does not only refer to metrics of attrition (13-b) (often a binary variable), but also to more continuous exposure metrics such as "average session length". These must be accompanied by a technical description how a metric like a "session" is defined (e.g., timeout after idle time) [1] (report under item 6a).

	1	2	3	4	5	
subitem not at all important	○	○	○	◉	○	essential

Does your paper address subitem 17a-i?

Copy and paste relevant sections from the manuscript (include quotes in quotation marks "like this" to indicate direct quotes from your manuscript), or elaborate on this item by providing additional information not in the ms, or briefly explain why the item is not applicable/relevant for your study

The results on this are presented in Huis In Het Veld JG, van Asch IFM, Willemse BM, Verkade PJ, Pot AM, Blom MM, Groot Zwaaftink RBM, Francke AL. Process Evaluation of Nurse-Led Online Self-Management Support for Family Caregivers to Deal With Behavior Changes of a Relative With Dementia (Part 1): Mixed Methods Study. J Med Internet Res. 2019 Oct 11;21(10):e13002. doi: 10.2196/13002.

17b) For binary outcomes, presentation of both absolute and relative effect sizes is recommended

Does your paper address CONSORT subitem 17b? *

Copy and paste relevant sections from the manuscript (include quotes in quotation marks "like this" to indicate direct quotes from your manuscript), or elaborate on this item by providing additional information not in the ms, or briefly explain why the item is not applicable/relevant for your study

Crude and adjusted analysis are presented in table 3 and 4. Observed mean scores are presented in figure 1, 2, 3 and 4.

18) Results of any other analyses performed, including subgroup analyses and adjusted analyses, distinguishing pre-specified from exploratory

Does your paper address CONSORT subitem 18? *

Copy and paste relevant sections from the manuscript (include quotes in quotation marks "like this" to indicate direct quotes from your manuscript), or elaborate on this item by providing additional information not in the ms, or briefly explain why the item is not applicable/relevant for your study

Not applicable.

18-i) Subgroup analysis of comparing only users

A subgroup analysis of comparing only users is not uncommon in ehealth trials, but if done, it must be stressed that this is a self-selected sample and no longer an unbiased sample from a randomized trial (see 16-iii).

	1	2	3	4	5	
subitem not at all important	○	●	○	○	○	essential

Does your paper address subitem 18-i?

Copy and paste relevant sections from the manuscript (include quotes in quotation marks "like this" to indicate direct quotes from your manuscript), or elaborate on this item by providing additional information not in the ms, or briefly explain why the item is not applicable/relevant for your study

Jouw antwoord

19) All important harms or unintended effects in each group

(for specific guidance see CONSORT for harms)

Does your paper address CONSORT subitem 19? *

Copy and paste relevant sections from the manuscript (include quotes in quotation marks "like this" to indicate direct quotes from your manuscript), or elaborate on this item by providing additional information not in the ms, or briefly explain why the item is not applicable/relevant for your study

Not applicable. There were no harms or unintended effects observed in this trial.

19-i) Include privacy breaches, technical problems

Include privacy breaches, technical problems. This does not only include physical “harm” to participants, but also incidents such as perceived or real privacy breaches [1], technical problems, and other unexpected/unintended incidents. “Unintended effects” also includes unintended positive effects [2].

	1	2	3	4	5	
subitem not at all important	○	◉	○	○	○	essential

Does your paper address subitem 19-i?

Copy and paste relevant sections from the manuscript (include quotes in quotation marks "like this" to indicate direct quotes from your manuscript), or elaborate on this item by providing additional information not in the ms, or briefly explain why the item is not applicable/relevant for your study

Not applicable. There were no harms or unintended effects observed in this trial.

19-ii) Include qualitative feedback from participants or observations from staff/researchers

Include qualitative feedback from participants or observations from staff/researchers, if available, on strengths and shortcomings of the application, especially if they point to unintended/unexpected effects or uses. This includes (if available) reasons for why people did or did not use the application as intended by the developers.

	1	2	3	4	5	
subitem not at all important	○	○	◉	○	○	essential

Does your paper address subitem 19-ii?

Copy and paste relevant sections from the manuscript (include quotes in quotation marks "like this" to indicate direct quotes from your manuscript), or elaborate on this item by providing additional information not in the ms, or briefly explain why the item is not applicable/relevant for your study

Qualitative feedback from participants is presented in Huis In Het Veld JG, van Asch IFM, Willemse BM, Verkade PJ, Pot AM, Blom MM, Groot Zwaaftink RBM, Francke AL. Process Evaluation of Nurse-Led Online Self-Management Support for Family Caregivers to Deal With Behavior Changes of a Relative With Dementia (Part 1): Mixed Methods Study. J Med Internet Res. 2019 Oct 11;21(10):e13002. doi: 10.2196/13002.

DISCUSSION

22) Interpretation consistent with results, balancing benefits and harms, and considering other relevant evidence

NPT: In addition, take into account the choice of the comparator, lack of or partial blinding, and unequal expertise of care providers or centers in each group

22-i) Restate study questions and summarize the answers suggested by the data, starting with primary outcomes and process outcomes (use)

Restate study questions and summarize the answers suggested by the data, starting with primary outcomes and process outcomes (use).

	1	2	3	4	5	
subitem not at all important	○	○	○	○	●	essential

Does your paper address subitem 22-i? *

Copy and paste relevant sections from the manuscript (include quotes in quotation marks "like this" to indicate direct quotes from your manuscript), or elaborate on this item by providing additional information not in the ms, or briefly explain why the item is not applicable/relevant for your study

"Online self-management support involving e-mail contacts with a specialist dementia nurse, videos and e-bulletins showed no significant difference in family caregivers' self-efficacy caregiver compared to online interventions not involving personal e-mail contacts. Furthermore, no measurable improvements could be found for the medium intervention involving online videos and e-bulletins compared to the minor intervention only involving e-bulletins.
In addition, no differences were found between the online intervention arms for the quality of the relationship between the person with dementia and the family caregiver and the occurrence of behavior changes. These results are contrary to our expectation that family caregivers who received e-mail support would be better assisted in dealing with and responding to changes in behavior and would therefore improve in terms of self-efficacy. We expected that increased self-efficacy and better response of the family caregiver would also have an effect on the person with dementia and would therefore result in less strain on the relationship, better interaction and an decrease in the occurrence of behavior changes. However, as no effect was found on self-efficacy was found, this could also explain why no effect could be detected on the secondary outcomes (quality of the relationship and the occurrence of behavior) in this study."

22-ii) Highlight unanswered new questions, suggest future research

Highlight unanswered new questions, suggest future research.

	1	2	3	4	5	
subitem not at all important	○	○	○	◉	○	essential

Does your paper address subitem 22-ii?

Copy and paste relevant sections from the manuscript (include quotes in quotation marks "like this" to indicate direct quotes from your manuscript), or elaborate on this item by providing additional information not in the ms, or briefly explain why the item is not applicable/relevant for your study

"However, some limitations of this study are worth mentioning. Firstly, in the power calculation, we had estimated a difference of 0.8 between the intervention arms to detect a significant effect of the major self-management support intervention compared to the other intervention arms. The estimated difference proved to have been an overestimate. The small sample size might therefore have played a part in the null findings for our hypothesis that the major intervention arm would have a greater effect on self-efficacy than the other intervention arms. We acknowledge that our study may have been underpowered for detecting an effect of the online self-management support intervention. For future studies, larger studies are required to establish the effectiveness of online self-management support interventions [34]. Secondly, due to the small sample size, we were unable to determine the effects on participants who actually used the intervention components. Instead, data of all included participants were analyzed. Future research should focus on which intervention components best fit specific family caregivers. It is important to determine the family caregivers who will benefit the most from additional online assistance in order to provide tailored personalized support. This will be more cost-effective, allowing nurses' support to be offered to the people who need it the most."

20) Trial limitations, addressing sources of potential bias, imprecision, and, if relevant, multiplicity of analyses

20-i) Typical limitations in ehealth trials

Typical limitations in ehealth trials: Participants in ehealth trials are rarely blinded. Ehealth trials often look at a multiplicity of outcomes, increasing risk for a Type I error. Discuss biases due to non-use of the intervention/usability issues, biases through informed consent procedures, unexpected events.

	1	2	3	4	5	
subitem not at all important	○	○	○	◉	○	essential

Does your paper address subitem 20-i? *

Copy and paste relevant sections from the manuscript (include quotes in quotation marks "like this" to indicate direct quotes from your manuscript), or elaborate on this item by providing additional information not in the ms, or briefly explain why the item is not applicable/relevant for your study

"Firstly, the process evaluation showed variation in the extent to which family caregivers made use of the various elements. Seventy-eight percent of the family caregivers in question used the opportunity of having e-mail contacts, 80% of the family caregivers clicked the links of one or more videos, but just 37% of all family caregivers clicked the links of at least one e-bulletin. Also, the use of e-mail contacts, videos and/or e-bulletins varied considerably within in each group. Therefore, the distinction between the three intervention arms became less, which makes it less likely to find statistically significant differences between the intervention arms. Low usage rates and differences in the use of online interventions are known problems [31, 32] which could explain why no positive effects were found in this study. "

21) Generalisability (external validity, applicability) of the trial findings

NPT: External validity of the trial findings according to the intervention, comparators, patients, and care providers or centers involved in the trial

21-i) Generalizability to other populations

Generalizability to other populations: In particular, discuss generalizability to a general Internet population, outside of a RCT setting, and general patient population, including applicability of the study results for other organizations

	1	2	3	4	5	
subitem not at all important	○	○	○	◉	○	essential

Does your paper address subitem 21-i?

Copy and paste relevant sections from the manuscript (include quotes in quotation marks "like this" to indicate direct quotes from your manuscript), or elaborate on this item by providing additional information not in the ms, or briefly explain why the item is not applicable/relevant for your study

"Firstly, in the power calculation, we had estimated a difference of 0.8 between the intervention arms to detect a significant effect of the major self-management support intervention compared to the other intervention arms. The estimated difference proved to have been an overestimate. The small sample size might therefore have played a part in the null findings for our hypothesis that the major intervention arm would have a greater effect on self-efficacy than the other intervention arms. We acknowledge that our study may have been underpowered for detecting an effect of the online self-management support intervention. For future studies, larger studies are required to establish the effectiveness of online self-management support interventions [34]."

21-ii) Discuss if there were elements in the RCT that would be different in a routine application setting

Discuss if there were elements in the RCT that would be different in a routine application setting (e.g., prompts/reminders, more human involvement, training sessions or other co-interventions) and what impact the omission of these elements could have on use, adoption, or outcomes if the intervention is applied outside of a RCT setting.

	1	2	3	4	5	
subitem not at all important	○	○	◉	○	○	essential

Does your paper address subitem 21-ii?

Copy and paste relevant sections from the manuscript (include quotes in quotation marks "like this" to indicate direct quotes from your manuscript), or elaborate on this item by providing additional information not in the ms, or briefly explain why the item is not applicable/relevant for your study

"Based on the findings of the process evaluation [30] we have two recommendations for future use of the intervention. Firstly, we recommend that nurses are instructed more explicitly and made more aware of the importance of the integrated use of the various elements (e-mail contacts, videos, e-bulletin) in the interventions. Secondly, for future use the intervention could involve more e-mail contacts."

OTHER INFORMATION

23) Registration number and name of trial registry

Does your paper address CONSORT subitem 23? *

Copy and paste relevant sections from the manuscript (include quotes in quotation marks "like this" to indicate direct quotes from your manuscript), or elaborate on this item by providing additional information not in the ms, or briefly explain why the item is not applicable/relevant for your study

"Trial Registration: Netherlands Trial Registry (NTR): NTR6237;
http://www.trialregister.nl/trialreg/admin/rctview.asp?TC=6237
(Archived by WebCite at http://www.webcitation.org/6v0S4fxTC)"

24) Where the full trial protocol can be accessed, if available

Does your paper address CONSORT subitem 24? *

Cite a Multimedia Appendix, other reference, or copy and paste relevant sections from the manuscript (include quotes in quotation marks "like this" to indicate direct quotes from your manuscript), or elaborate on this item by providing additional information not in the ms, or briefly explain why the item is not applicable/relevant for your study

"For more details, the readers are referred to the full intervention protocol, available in https://www.nivel.nl/sites/default/files/bestanden/Intervention-procotol-self-management-support-dementia.pdf. "

25) Sources of funding and other support (such as supply of drugs), role of funders

Does your paper address CONSORT subitem 25? *

Copy and paste relevant sections from the manuscript (include quotes in quotation marks "like this" to indicate direct quotes from your manuscript), or elaborate on this item by providing additional information not in the ms, or briefly explain why the item is not applicable/relevant for your study

"Funding: This study is part of the research program called 'NurseSMS- Nurses Self-management Support for people facing progressive incurable diseases' (2014–2019), which is funded by ZonMw (Netherlands Organization for Health Research and Development), program "Tussen Weten en Doen"."

X27) Conflicts of Interest (not a CONSORT item)

X27-i) State the relation of the study team towards the system being evaluated

In addition to the usual declaration of interests (financial or otherwise), also state the relation of the study team towards the system being evaluated, i.e., state if the authors/evaluators are distinct from or identical with the developers/sponsors of the intervention.

	1	2	3	4	5	
subitem not at all important	○	○	○	○	○	essential

Does your paper address subitem X27-i?

Copy and paste relevant sections from the manuscript (include quotes in quotation marks "like this" to indicate direct quotes from your manuscript), or elaborate on this item by providing additional information not in the ms, or briefly explain why the item is not applicable/relevant for your study

"Conflicts of interest: The authors have no conflicts of interest to declare."

About the CONSORT EHEALTH checklist

As a result of using this checklist, did you make changes in your manuscript? *

- ○ yes, major changes
- ○ yes, minor changes
- ◉ no

What were the most important changes you made as a result of using this checklist?

Jouw antwoord

How much time did you spend on going through the checklist INCLUDING making changes in your manuscript *

2,5 hours

As a result of using this checklist, do you think your manuscript has improved? *

- ○ yes
- ◉ no
- ○ Anders:

Would you like to become involved in the CONSORT EHEALTH group?

This would involve for example becoming involved in participating in a workshop and writing an "Explanation and Elaboration" document

- ○ yes
- ◉ no
- ○ Anders:

Any other comments or questions on CONSORT EHEALTH

Jouw antwoord

STOP - Save this form as PDF before you click submit

To generate a record that you filled in this form, we recommend to generate a PDF of this page (on a Mac, simply select "print" and then select "print as PDF") before you submit it.

When you submit your (revised) paper to JMIR, please upload the PDF as supplementary file.

Don't worry if some text in the textboxes is cut off, as we still have the complete information in our database. Thank you!

Final step: Click submit !

Click submit so we have your answers in our database!

Verzenden

Verzend nooit wachtwoorden via Google Formulieren.

Deze content is niet gemaakt of goedgekeurd door Google. Misbruik rapporteren - Servicevoorwaarden - Privacybeleid

Google Formulieren
